# Factors Associated with Divergent Introgression Outcomes from *Wolbachia*-Based Interventions in Rio de Janeiro, Brazil

**DOI:** 10.64898/2026.10.16.26365711

**Published:** 2026-10-21

**Authors:** Elisabeth Nelson, Paloma M. Cárcamo, Gabriel Ribeiro dos Santos, Gabriel Sylvestre Ribeiro, Thiago Rodrigues da Costa, Ilaria Dorigatti, Henrik Salje, Luciano A. Moreira, Derek A.T. Cummings, Albert I. Ko

**Affiliations:** 1 Department of Epidemiology of Microbial Diseases, Yale School of Public Health. P.O. Box 208034, New Haven, CT 06520-8034, United States.; 2 Departments of Epidemiology and Biomedical Engineering, Johns Hopkins University, 615 N Wolfe St, Baltimore, MD 21205, United States.; 3 World Mosquito Program Brazil. Avenida Brasil, 4.036 - Campus Expansão da Fundação Oswaldo Cruz - 10° andar - sala 1014, Manguinhos - Rio de Janeiro, CEP 21040-900, Brazil.; 4 MRC Centre for Global Infectious Disease Analysis, School of Public Health, Imperial College London, 90 Wood Lane, London, W12 OBZ, UK; 5 Department of Genetics, University of Cambridge. Downing Street, Cambridge, CB2 3EH, United Kingdom.; 6 Emerging Pathogens Institute and Department of Biology, University of Florida, 2055 Mowry Rd, Gainesville, FL 32610, United States.; 7 Instituto René Rachou – Fiocruz Minas, Av. Augusto de Lima, 1715 – Barro Preto, Belo Horizonte, Minas Gerais, 30190-022, Brazil; 8 Instituto Gonçalo Moniz, Fundação Oswaldo Cruz, Rua Waldemar Falcão, 121, Candeal, Salvador, Bahia, CEP 40296-710, Brazil.

## Abstract

*Wolbachia*-based interventions were less effective in achieving introgression in Rio de Janeiro than other settings. Reduced introgression occurred in locations where dengue was historically high, raising concerns of inequity. We examined factors affecting *Wolbachia* introgression in Rio through trajectory modeling, spatiotemporal Bayesian models, and *Ae. aegypti* abundance estimation. During the intervention (2017–2020), *Wolbachia* introgression was 26% (median; IQR,14–44%), with four distinct trajectories. Despite receiving more releases, densely populated areas with low socioeconomic status had lower introgression. In spatiotemporally explicit multivariable regressions, higher *Ae. aegypti* abundance, temperature, population density and lower socioeconomic status were associated with reduced introgression. Improved characterization of *Ae. aegypti* abundance and other structural/environmental predictors of introgression could be used to calibrate release doses to ensure equitable benefits from *Wolbachia* interventions.

The global burden of dengue and other arboviral diseases has been increasing worldwide, highlighting the need for effective interventions to control transmission (1,2). Transinfection of the endosymbiotic bacteria *Wolbachia pipientis* in *Aedes aegypti* mosquitoes, the main vector for dengue, can block transmission of dengue virus (DENV) and other arboviruses within the mosquito (3,4). *Wolbachia* infection induces cytoplasmic incompatibility in *Ae. aegypti*, meaning that uninfected females that mate with infected males produce infertile eggs, while infected females will have viable eggs. This phenomenon allows for *Wolbachia* infection to rapidly and sustainably introgress and remain stable in *Ae. aegypti* populations over long periods if sufficient numbers of *Wolbachia* infected mosquitoes are released into an area resulting in increased frequency-dependent fitness of infected females compared to uninfected mosquitoes (5,6).

The potential of *Wolbachia*-based interventions to reduce arboviral disease transmission has led to several studies and interventions. A randomized trial conducted in Yogyakarta found that after achieving high levels of introgression, i.e. prevalence, of the *w*Mel strain of *Wolbachia* (~98.5%), dengue risk was reduced by approximately 77% (7). However, in other settings, such as Rio de Janeiro in Brazil, releases have been less effective at achieving high levels of introgression and reducing risk of arboviral disease (8). In addition, the effectiveness of the intervention in this setting was found to be highly heterogeneous across release areas. Areas with historically higher rates of dengue and chikungunya incidence achieved lower levels of introgression, and therefore, lower reductions in arboviral disease. Our study aimed to characterize the variability in introgression in Rio de Janeiro and quantify the impact of operational, meteorological, socioeconomic and environmental factors on introgression to inform optimization of *w*Mel releases and achieve equitable introgression.

## Implementation of *w*Mel releases and introgression monitoring in Rio de Janeiro

The World Mosquito Program (WMP) released adult *w*Mel-positive *Ae. aegypti* in an 86.8 km^2^ area in northwest Rio de Janeiro (Figure 1A) between August 2017 and March 2020 (8,9). The area was divided into five intervention sites, which followed different release schedules. Tubes containing an average of 350 mosquitoes were emptied every 50m by vehicle or by foot. *w*Mel introgression was monitored using a network of 1168 BG-traps, which were collected biweekly. Up to 10 *Ae. aegypti* per trap were tested for *w*Mel using a validated qPCR assay. Introgression was estimated as the proportion of tested mosquitoes that were positive for *w*Mel (i.e. *w*Mel prevalence among host-seeking adult females captured in BG-Sentinel traps). If a trap was found to have consistently low levels of introgression, releases were continued in the surrounding area, leading to differing lengths of the intervention across the study area (Figure 1D).

By the end of the intervention, there was marked heterogeneity in the cumulative dose of released *w*Mel *Ae. aegypti* across the intervention area (median 10,428, interquartile range [IQR] 6,854–14,740 mosquitoes per 0.01km^2^) (Figure 1B), as well as in the cumulative dose of released *w*Mel *Ae. aegypti* per inhabitant (median 79.10, IQR 21.26–171.57 mosquitoes per inhabitant in 0.01km^2^) (Figure 1C). Areas with lower cumulative dose per inhabitant had lower income levels and less impervious surfaces (Tables S1, S2).

## Characterization of trap-level introgression trajectories through time

Mean *w*Mel introgression throughout the study period was low in most traps (median 0.26, IQR 0.14–0.44) (Figure 2A). We conducted group-based trajectory modeling on spline-smoothed trap-level introgression trajectories and identified four clusters of distinct patterns of introgression trajectories (Figures 2B–C). We observed an inverse association between introgression and cumulative releases at the cluster level, suggesting that continued releases of the same dose over time may not be enough to ensure introgression. The cluster with the most successful introgression (Cluster 1) had received the lowest cumulative dose of *w*Mel *Ae. aegypti* releases by area, while the cluster with lowest mean introgression (Cluster 4) had received the highest cumulative releases (Figure 2D).

To explore factors that could explain the observed differences in introgression trajectories, we obtained total *Ae. aegypti* counts from each trap and extracted information on socioeconomic and environmental covariates from a 100m radius around each trap. We found that clusters with highest introgression had lower mosquito abundance as measured by *Ae. aegypti* trap counts (Figure 2D). We also observed that clusters with higher levels of introgression had lower human population density, higher income, less impervious surfaces and more green spaces (Table S3).

## Quantification of spatiotemporal factors influencing trap-level introgression

To formally test the influence of operational, meteorological, socioeconomic, environmental, and entomological variables on introgression, we developed a spatiotemporally explicit multivariable model using the Integrated Nested Laplace Approximation (INLA) method of Bayesian inference. Using trap-level *w*Mel prevalence as the outcome, we performed a hierarchical model fitting process, successively testing a wide range of covariates for association with the outcome (Table S4). Covariates significantly associated with the outcome which improved the model fit were included as either linear or non-linear depending on their observed association with the outcome, using univariate generalized additive models with a cubic regression spline to infer linearity (Figure S1). We expect environmental factors to impact different stages of the mosquito life cycle and may have delayed effects on abundance and introgression. To account for this, we assessed different temporal lags and selected the ones significantly correlated with the outcome in univariate models to use in the model (Figure S2 and S3).

We found that measures of *Ae. aegypti* abundance, previous introgression, meteorological conditions, socioeconomic indicators, and metrics of the built environment, were associated with lower introgression (Figure 3). Several of these factors are associated with larger *Ae. aegypti* populations. To account for potential contamination of introgression estimates during and directly after releases, we included terms related to whether releases occurred and the number of mosquitoes released in the previous week, which controls for the potential recapture of released *w*Mel -infected mosquitoes. We found increasing *Ae. aegypti* abundance had a negative association with introgression. We found the highest quintile of mosquito trap counts (27 *Ae. aegypti*) one- and two-weeks prior were associated with a 5% (95% CrI 2–8%) decrease in introgression as compared to the median trap count (4 *Ae. aegypti*). Increasing previous introgression one- and two-weeks prior were strongly associated with an increase in introgression by 76% (95% CrI 68–84%) and 34% (95% CrI 29–40%) respectively. It is likely that this autocorrelation captures the frequency-dependent fitness effects associated with *w*Mel introgression (cytoplasmic incompatibility and maternal inheritance). Notably, in a model excluding the lagged-introgression and abundance terms (Figure S4), the socioeconomic, environmental, and meteorological associations persisted with the same direction of effect. This indicates that these effects are not artifacts of the autoregressive structure; they are identified primarily from spatial (between-location) variation, which the temporal autoregressive term does not absorb.

Investigating the effects of temporally-varying meteorological factors, we found increasing minimum weekly temperature four weeks prior were negatively associated with introgression. Observations in the lowest quintile of minimum temperatures, around 15.9°C, had 29% (95% CrI 10–51%) more introgression than those with the median minimum temperature (20°C), while the highest quintile of minimum temperature, around 24°C, had a 20% (95% CrI 8–31%) negative effect relative to the median. Increasing minimum temperature four weeks prior is beneficial for the development of the previous generation of *Ae. aegypti* and for the egg and larval stages of the current adult population, having an indirect association with larger current *Ae. aegypti* populations (10–13). These temperature effects appear to be driven by seasonal temperature changes, rather than weather extremes (Figure S5). This suggests that introgression is less effective under conditions associated with larger wildtype *Ae. aegypti* populations, such as warmer seasons (5,14–16). Higher temperatures may also lead to lower introgression due to the degradation of *w*Mel *Wolbachia* in *Ae. aegypti* eggs at higher temperatures (17–20). The covariate selection process is fully documented in Table S4, and the consistency of associations across multiple sensitivity analyses (varying spatial scale, temporal autocorrelation structure, inclusion/exclusion of abundance terms, and restriction to non-release periods) provides empirical reassurance that the reported associations are not artifacts of the selection procedure.

Additionally, spatially varying factors associated with greater percentages of built environment and lower socioeconomic status (SES) were also negatively associated with introgression. Observations in the highest population density of around 889.2 inhabitants per 100m radius had 8% (95% CrI 1–14%) less introgression than the median population density (366.3 inhabitants per 100m radius). Population density is also often an indicator of *Ae. aegypti* abundance, with higher population density found to be associated with increased *Ae. aegypti* populations in other settings (21). Of note, *Ae. albopictus* is present in Rio de Janeiro but is largely restricted to peri-urban and forested areas rather than the dense urban neighborhoods covered by our study, making interspecific competition an unlikely major driver of the observed heterogeneity in introgression. Similarly, lowest income quintile (R$483.4 per month), had 12% (95% CrI 3–20%) less introgression than those with median income (R$964.5), whereas those in the second highest quintile (R$1349.1 per month), had 12% (95% CrI 5–20%) higher introgression than the median. Lower mean monthly income and other indicators of lower SES have been found to be associated with increased numbers of mosquito breeding sites and increased mosquito-human interactions (22). Observations at the highest elevation, around 41.3m, had 13% (95% CrI 5–20%) less introgression than those with median elevation (13.2m). In Rio de Janeiro, urban informal settlements are located at higher elevations, thus SES declines with increasing elevation. Observations in the highest impervious surface quintile, of 90.8%, had 8% (95% CrI 1–14%) less introgression than those with the median impervious surface (72.4%).

Introgression was better achieved in places with intermediate levels of impervious surfaces. Places with low levels of impervious surface could be less hospitable habitats for *Ae. aegypti* populations, while places with high levels of impervious surfaces could facilitate the proliferation of cryptic breeding sites and the presence of larger *Ae. aegypti* populations (13). Details on the fit of spatial and temporal autoregressive terms for both models and results from sensitivity analyses can be found in the supplements (Figures S6–S13), which yielded trends consistent with our main models.

Despite the trends in factors negatively associated with introgression being associated with larger *Ae. aegypti* populations, including *Ae. aegypti* abundance in the model does not entirely account for their effects. We separately assessed models that did not include mosquito monitoring data and used only information that could be collected prior to a *w*Mel release intervention and readily measured in many locales, i.e. meteorological conditions, socioeconomic indicators and metrics of the built environment (Figure S4). Comparison of the model results showed inclusion of *Ae. aegypti* abundance and previous *w*Mel introgression yielded a slight dilution in the effects of the meteorological, environmental, and socioeconomic factors. However, these factors still showed significant effects on introgression, suggesting that *Ae. aegypti* population size partially mediates the influence of these effects, but does not entirely account for them. Therefore, these factors may be acting independently through mechanisms outside of wildtype population size, in addition to their effects on population size. We note that our models use the extrapolated *w*Mel-positive count with an offset for the total *Ae. aegypti* captured, rather than the raw prevalence, so the inverse abundance association is unlikely to arise mechanically from a shared prevalence denominator; we therefore interpret it as reflecting reduced establishment rather than denominator inflation, while acknowledging that a residual mechanical contribution cannot be fully excluded. Additionally, it may also be due to trap captures imprecisely measuring *Ae. aegypti* populations, either due to sparse spatial sampling or variation in attractiveness of traps in different locations. We therefore investigated the potential use of more accurate measures of abundance.

## Prediction of introgression utilizing baseline entomological observations

We explored predictors of introgression that could be measured at the onset of the releases. To obtain a more precise estimate of *Ae. aegypti* abundance in the release areas, we used mark release recapture methods traditionally used for abundance measurements in ecology (23). We calculated the Fisher-Ford (FF) Index assuming the ratio of *w*Mel-infected to wild-type *Ae. aegypti* captured in traps reflected the ratio of *w*Mel mosquitoes released to the ambient wild-type population (23). We could only calculate this index for the first trap collection and if the collection occurred 36 days or less after the first release, when the majority of captured *w*Mel-positive mosquitoes are expected to have been released and not introgressed. We found a negative association between the baseline *Ae. aegypti* abundance and mean *w*Mel introgression. Conversely, the ratio of *w*Mel mosquitoes released to abundance had a positive association with mean *w*Mel introgression (Figure 4). Both associations were consistent across assumed daily survival probabilities from 0.75 to 0.90 (Figure S14). This release-to-abundance ratio was associated with whether mean introgression throughout the intervention was above 20% (Figure S15–S16). In addition, we found that higher percentages of green space (odds ratio [OR] 1.47 per standard deviation [SD] increase) and higher mean income of head of household (OR 1.52 per SD) were positively associated with mean *w*Mel introgression. In contrast, a higher percentage of impervious surfaces was negatively associated with mean introgression (OR 0.63 per SD).

These findings suggest that baseline *Ae. aegypti* abundance, the relative dose of *w*Mel *Ae. aegypti* released compared to existing mosquito populations, landscape features and socioeconomic characteristics determine the success of the intervention. *Wolbachia* introgression has been described to achieve population replacement only if the proportion of *Wolbachia*-positive mosquitoes exceeds a certain threshold; introgression declines to zero otherwise (6). In our study setting, areas with high mosquito abundance may have not received large enough *w*Mel *Ae. aegypti* doses per release to exceeding this threshold and achieving population replacement, even if releases continued over a longer time. More robust estimates of baseline *Ae. aegypti* abundance will be helpful in confirming these associations and to better inform planning of releases. These baseline estimates rest on a limited number of traps (n = 85) and assume a fixed daily survival probability; immigration of wild-type mosquitoes from untreated areas and higher catchability of recently released compared to wild mosquitoes would both lead to underestimation of the FF index and therefore overestimation of the release-to-abundance ratio. We therefore present this analysis as hypothesis-generating and requiring confirmation through additional analyses.

## Limitations

There are several limitations inherent to our study. The number of mosquitoes caught during each trapping event was highly variable and introgression estimates were often based on the results of testing fewer than ten mosquitoes, which could lead to imprecise estimates. To address this, we smoothed introgression estimates, weighing by the number of mosquitoes tested, to account for differing levels of precision in different trapping events and used extrapolated *w*Mel counts as the outcome in our models.

Furthermore, our selection of covariates was constrained by the availability of data. We did not include spatially varying temperature and precipitation because there is only one Brazilian National Institute of Meteorology weather station in the study area. Nevertheless, given the small size of the study area, we do not expect significant spatial variability in these meteorological factors. Additionally, this weather station does not measure humidity, which has been shown to impact *Ae. aegypti* survival (24,25). We used BG-trap captures as a proxy for *Ae. aegypti* abundance, which may be underestimated in high-density, low-SES locations due to competition with human hosts for trap attractants. We did not include information on vector control practices or insecticide use due to lack of data availability. A change in the larvicide used in routine larval source management has been found in a recent paper to be associated with declines in long-term introgression stability in part of the intervention area, potentially due to *Wolbachia*-related fitness costs. (26). However, in our study, we found introgression levels remained stable through time at both high and low levels. Nonetheless, we included most factors shown in the literature to have a potential effect on *Ae. aegypti* abundance and/or *w*Mel introgression. Our socioeconomic indicators were derived from the 2010 census and thus predate the intervention by several years, and elevation served in part as a proxy for socioeconomic status; since these indicators are correlated with *Ae. aegypti* abundance and the built environment, the equity-relevant associations should be interpreted as describing the joint structural context of low introgression rather than as isolating an independent causal effect of socioeconomic status. The 2010 census was used as it is the last Brazilian census with complete data; preliminary results from the 2022 census are available and socioeconomic metrics in Rio de Janeiro are very highly correlated between the two datasets. The mediation analysis (Figure S4) indicates SES effects are only partly explained by mosquito abundance. Additionally, release-batch quality (e.g., longevity, mating competitiveness) was not systematically recorded and represents could contribute to heterogeneity in establishment.

Finally, we estimated mosquito abundance for each trap at different calendar times, based on when initial releases and trapping began. Some of the observed differences in abundance may reflect seasonal fluctuations in mosquito populations. However, we calculated the ratio of the initial release dose to the existing mosquito population at the same calendar time, enabling us to assess intervention dosing regardless of the season.

## Conclusions

Despite many obstacles, WMP was able to implement the *w*Mel intervention in Rio de Janeiro by working with communities and local authorities throughout the study area, including less accessible, informal settlement areas. However, we found cumulative releases of *w*Mel varied significantly by area and by inhabitant and there was heterogeneity in introgression throughout the study area. In more recent release programs in Brazil, WMP has determined release doses using human population density rather than area. In Belo Horizonte, as part of the EVITA Dengue Trial, they aimed to release 88–100 *w*Mel *Ae. aegypti* per person in the first 16 weeks, targeting more homogenous introgression (27). The areas with the most successful introgression in Rio de Janeiro were higher in the socioeconomic status and achieved higher introgression with less *w*Mel released by area, highlighting inequities in the intervention outcome. After more formally testing with spatiotemporal models, we identified that lower socioeconomic status, increased built environment, and higher *Ae. aegypti* abundance were associated with lower introgression. Furthermore, we found that the ratio of mosquitoes released to baseline mosquito abundance strongly correlated with mean introgression levels, suggesting its potential utility as a planning metric. Accounting for existing *Ae. aegypti* populations when planning releases will play a key role in ensuring successful, equitable, and sustainable *Wolbachia* interventions.

## Supplementary Material

1

Materials and methods

Figures S1 to S16

Tables S1 to S4

## Figures and Tables

**Figure 1.**
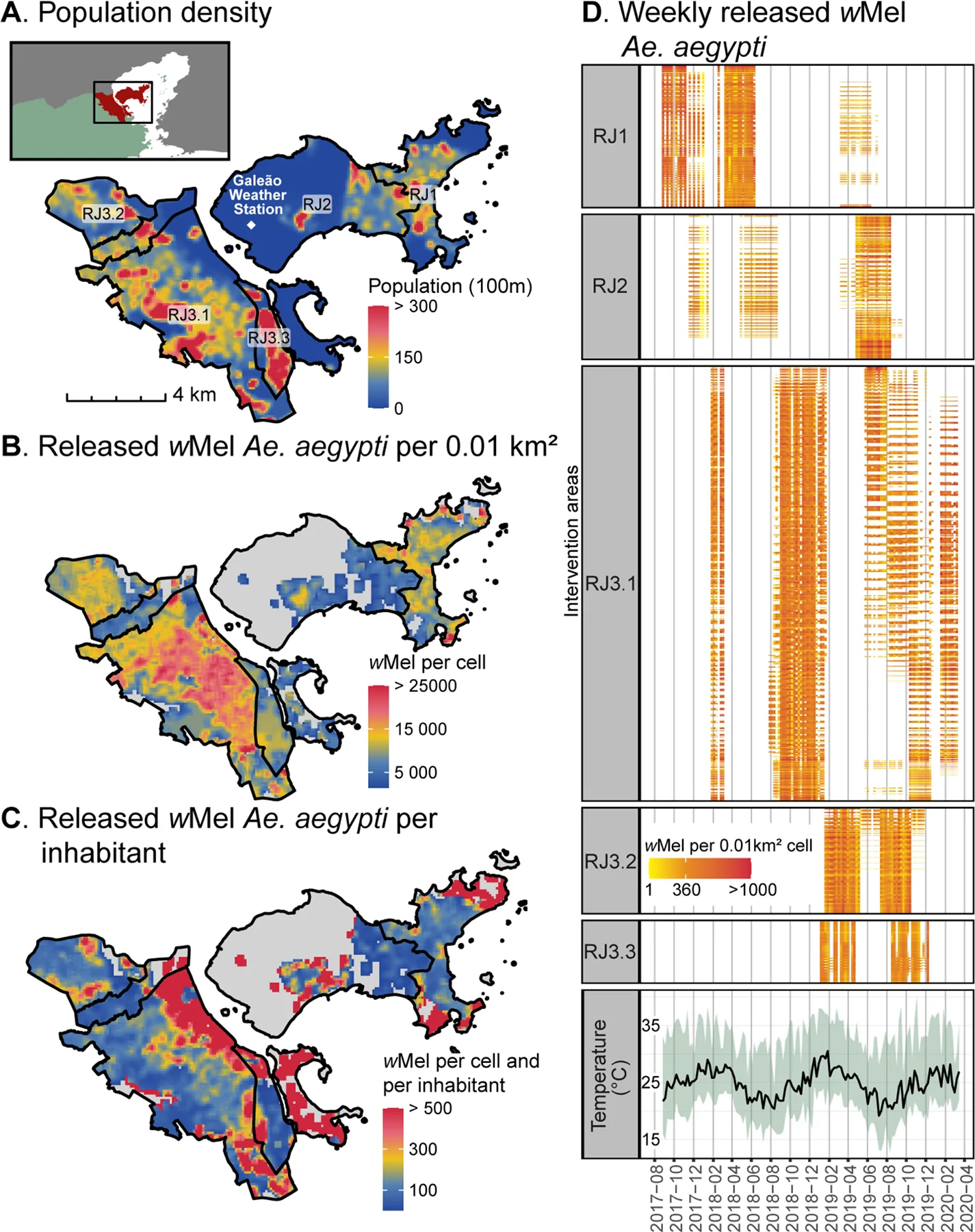
Roll-out of *w*Mel intervention in Rio de Janeiro, 2017–2020. (A, B, C) Maps of population density and releases. The intervention was not implemented in areas shaded in gray. (D) Release schedule of each intervention area. Rows represent 0.01km^2^ cells. Daily temperature (mean, minimum and maximum) in degrees Celsius measured in Galeão Station, located in RJ2.

**Figure 2.**
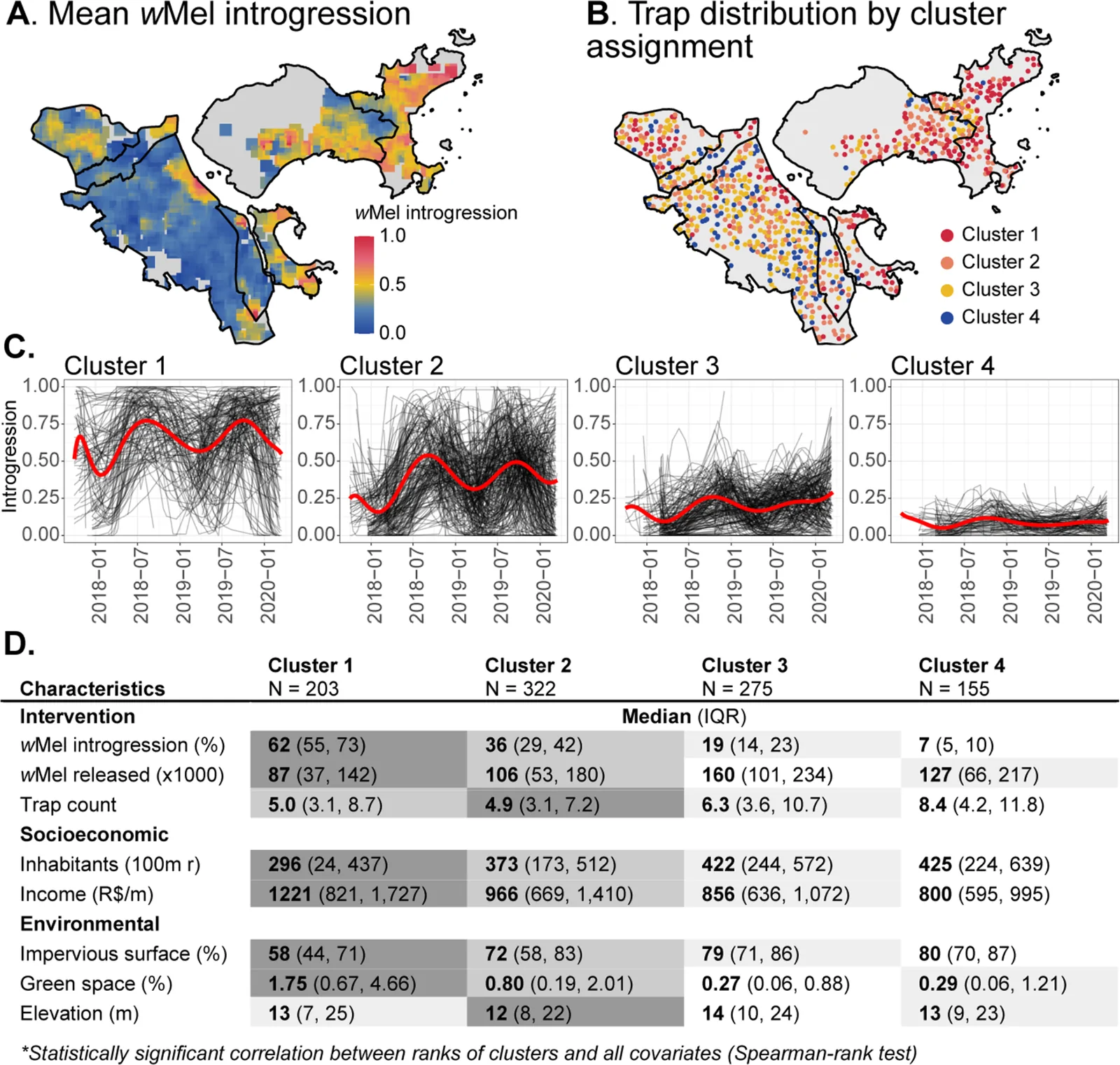
Group-based multivariate trajectory modeling (GBMT) of *w*Mel introgression trajectories. (A) Map of mean *w*Mel introgression throughout the study period. Gray areas did not have traps. (B) Map of spatial distribution of 955 traps included in GBMT. (C) Smoothed introgression trajectories of individual traps stratified by cluster. Red lines are the estimated trajectory for each cluster. (D) Characteristics of intervention, socioeconomic, and environmental factors in 100m radius of each trap. Boxes are shaded according to each cluster’s rank for a specific characteristic.

**Figure 3.**
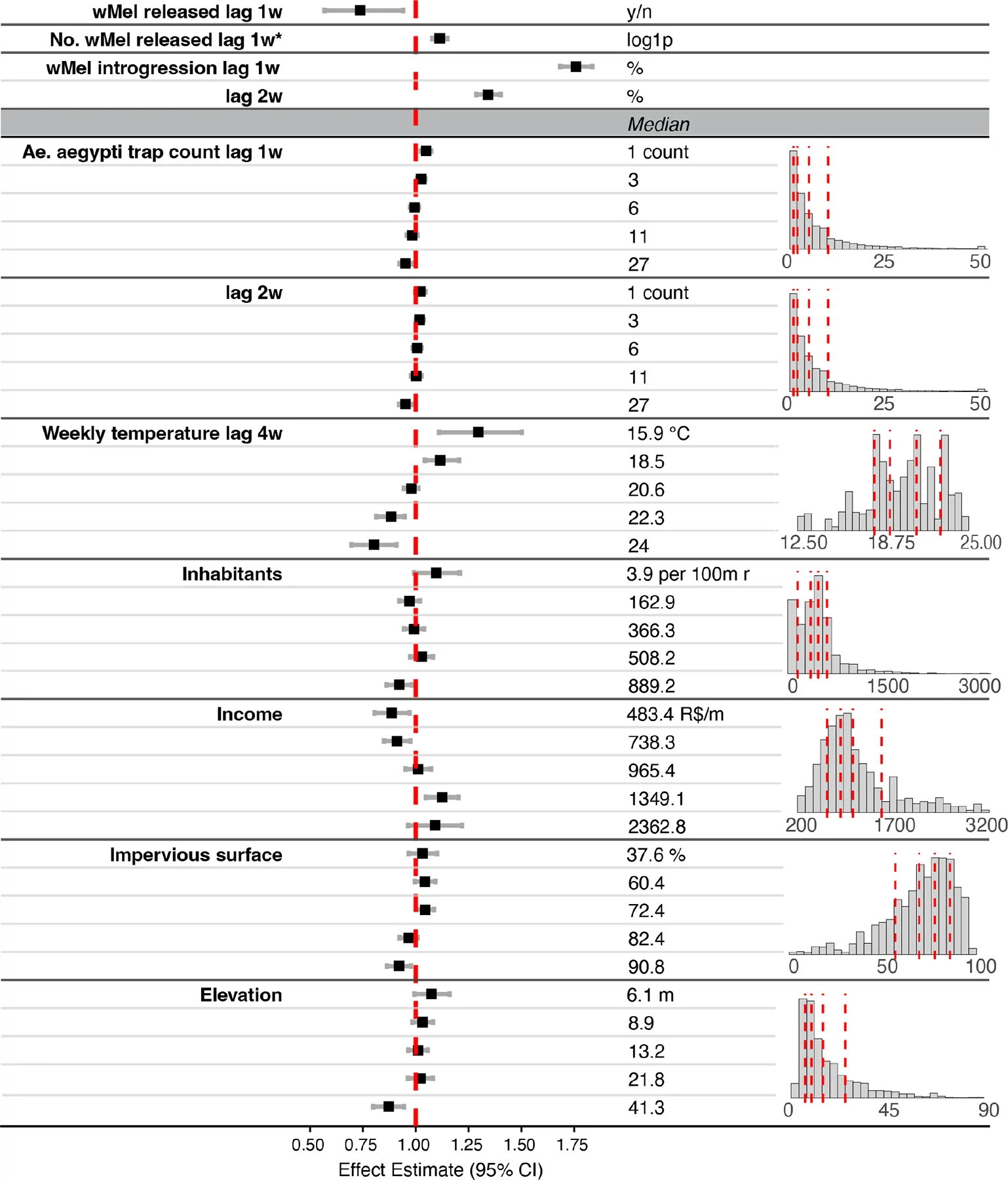
Spatiotemporal analysis of factors measured during the intervention associated with *w*Mel introgression (Conditional R^2^=0.76). The outcome is extrapolated *w*Mel count (*w*Mel prevalence*Ae. aegypti caught). Terms above the gray ribbon were included as linear and those below as non-linear. Non-linear effects were run as quintiles, depicted in the distribution plots, with red lines marking the boundaries, and medians and units of measurement in the text column. Data was aggregated by week and scaled to the 100m radius surrounding traps. ‘*w*Mel released lag 1w’ is a binary term, indicating whether releases occurred; ‘No. *w*Mel released lag 1w*’ is the log(1+x) or log1p number of mosquitoes released; ‘*w*Mel introgression lag 1w, 2w’ is the percent *w*Mel-positive mosquitoes; ‘Daily precipitation lag 6w’ is the mean daily precipitation, in centimeters; ‘*Ae. aegypti* trap count lag 1w, 2w’ are measures of *Ae. aegypti* abundance, distribution tails not depicted; ‘Weekly temperature lag 4w’ is the minimum weekly temperature, in Celsius; ‘Inhabitants’ is the population density; ‘Income’ is the mean income in R$ for persons over 10; ‘Impervious surface’ is the percent area comprised of water-impervious surfaces; ‘Elevation’ is in meters.

**Figure 4.**
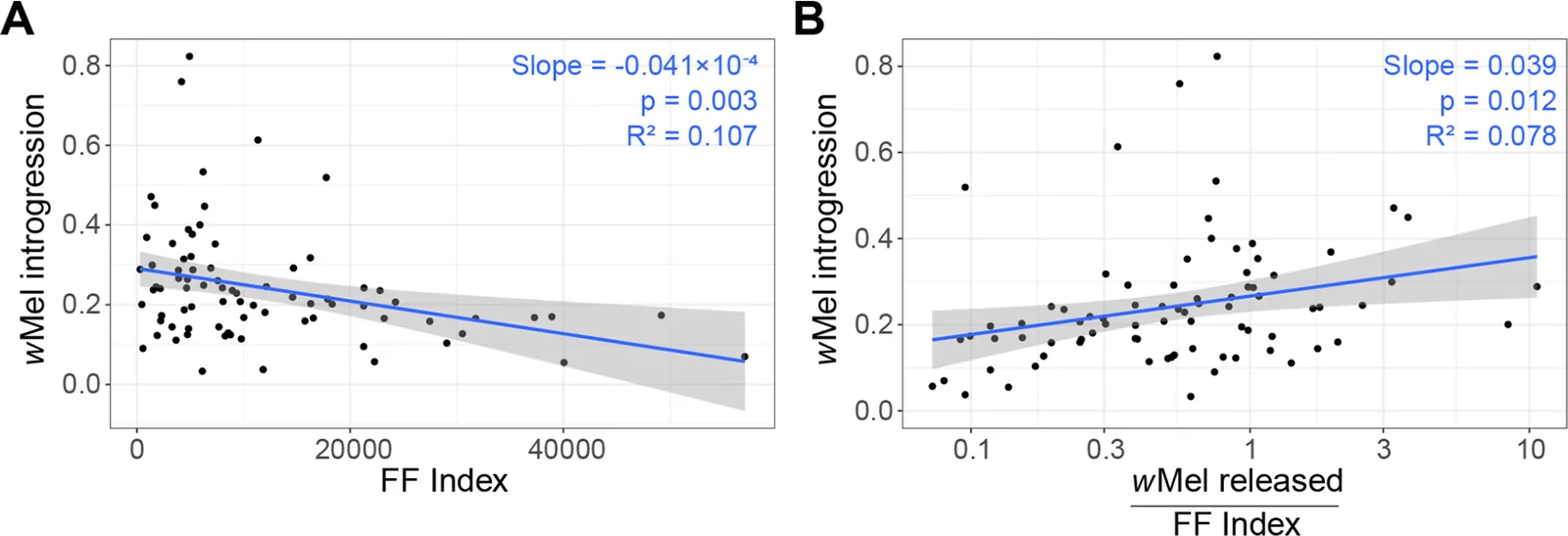
Baseline abundance estimates and introgression in traps with 9–36 days between first release and first trapping event. (A) Fisher-Ford (FF) Index and (B) ratio of wMel released (mean of releases before first trapping event) to Fisher-Ford Index versus mean *w*Mel introgression (log-transformed) for 85 traps; blue lines show linear regression trend with 95% CI. Blue text indicates regression slope, significance, and explained deviance (R^2^).

## Data Availability

All code and de-identified data used in these analyses are available at https://github.com/ko-laboratory/wmel-rj.
