# Supplementary material for: Factors Associated with Divergent Introgression Outcomes from Wolbachia-Based Interventions in Rio de Janeiro, Brazil
Source: medRxiv. 2026 Oct 21:2026.10.16.26365711. Preprint. [Version 1] doi: 10.64898/2026.10.16.26365711 (PMC13738876; doi:10.64898/2026.10.16.26365711)
Supplement: Supplementary file 1 [file NIHPP2026.10.16.26365711V1-supplement-1.pdf]

medRxiv preprint doi: https://doi.org/10.64898/2026.10.16.26365711; this version posted October 21, 2026. The copyright holder for this preprint (which was not certified by peer review) is the author/funder, who has granted medRxiv a license to display the preprint in perpetuity.
It is made available under a CC-BY 4.0 International license .

Supplementary Materials

Materials and Methods

Setting

Rio de Janeiro, the second largest city in Brazil, is a tropical city located in the Southeastern state of Rio de Janeiro (28). Covering 1,255km^2, with a population of almost 6.7 million inhabitants, the city is characterized by varying urban landscapes. Elevation within the city ranges from 0m to 1,022m above sea level, with poorer areas generally located atop the steep, less accessible mountains (29). Temperatures range from around 21℃ in the winter/dry season to around 30℃ in the summer/wet season. Precipitation ranges from an average of 5cm in the winter/dry season to an average of 17cm in the summer/wet season.

Releases

World Mosquito Program Brazil performed releases of *w*Mel *Ae. aegypti* mosquitoes from August 2017 through March 2020 and in five zones of Rio de Janeiro, covering a total area of 86.8 km^2 and a population of roughly 890,000. Releases were performed in three phases: an initial establishment phase of

medRxiv preprint doi: https://doi.org/10.64898/2026.10.16.26365711; this version posted October 21, 2026. The copyright holder for this preprint (which was not certified by peer review) is the author/funder, who has granted medRxiv a license to display the preprint in perpetuity.
It is made available under a CC-BY 4.0 International license .

weekly releases for a period of 8-16 weeks, followed by up to two consolidation phases of weekly to biweekly releases performed until *w*Mel prevalence reached >60% in the area or the end of the intervention period. Approximately 350 adult *w*Mel *Ae. aegypti* mosquitoes were released at 50m intervals throughout release areas. Where possible, releases occurred using vehicles, but in less accessible areas, releases were performed on foot. Regulatory approval for field releases was obtained from the National Research Ethics Committee (CONEP, CAAE 02524513.0.1001.0008), as described in previous publications (9).

Trapping

Monitoring of *Ae. aegypti* abundance and *w*Mel prevalence was conducted using a network of 1,168 BG-Sentinel traps, placed at even intervals approximately every 250m and up to 16 traps per km^2 (8,30). Traps were collected biweekly. All mosquitoes captured were morphologically identified to the species level and a random sample of up to 10 *Ae. aegypti* were tested individually for *w*Mel using quantitative PCR.

Covariates

We compiled data on meteorological, socioeconomic/demographic, and environmental factors potentially influencing *w*Mel introgression in the study area and time period from several sources. Meteorologic data was collected from the Brazilian National Institute of Meteorology, which maintains one meteorological station in the study area on Ilha do Governador (31). Hourly minimum, maximum, and mean temperatures (°C) and mean daily precipitation (cm) from the Galeão weather station were aggregated by week. Socioeconomic data was collected from the 2010 Brazilian Census, including several indicators of income, electricity, literacy, waste management, and piped water access at the census-tract level (32). Population density was collected from WorldPop, which uses a random forest regression tree-based mapping approach to integrate census data with other spatial datasets related to human populations to provide finer population density estimates, at 100x100m scale (33). Elevation (m) and impervious surface (%) were collected at the 30 x 30m scale from LandSat, NASA and the U.S. Geological Survey's satellite imagery database (34). Urban Green Space data at the 10x10m scale was collected from Google Earth Engine's Sentinel-2 database (35).

Descriptive Analysis

To understand the spatiotemporal heterogeneities in the release, trapping, and covariate data, as well as to assess differences in cumulative *w*Mel releases by area, we divided the study area into 11,140 100x100m cells. In each grid cell, we calculated the cumulative number of *w*Mel *Ae. aegypti* released by cell (area) and by person (inhabitants in each cell). We divided the cells into quartiles by the number of mosquitoes released by area and by person. We then described the distribution of the covariates in each quartile to understand trends in program roll-out. We also compared the number of mosquitoes released by area and by person with the average *w*Mel introgression in that cell.

Introgression Trajectory Modeling

To understand trends in *w*Mel introgression at a finer scale, we analyzed individual trap-level introgression through time. Given the zero- and one-inflated nature of the trap-level data due to variations in the number of total *Ae. aegypti* caught through time, we smoothed the trap-level introgression trajectories through time using generalized additive models weighted by the total number of *Ae. aegypti* captured at each time point. To create clusters of introgression trajectories through time, we conducted group-based multivariate trajectory modeling (GBMT) on the trap-level smoothed data using the 'gbmt' R package (36). We used model-based information criterion scores and interpretability of clustering, i.e. balance of number of traps in each cluster, to select the best number of clusters. We then extracted covariate data from a 100m radius around each trap and described covariate characteristics by cluster (37). We tested varying radii measures as a sensitivity analysis (**Table S3**).

medRxiv preprint doi: https://doi.org/10.64898/2026.10.16.26365711; this version posted October 21, 2026. The copyright holder for this preprint (which was not certified by peer review) is the author/funder, who has granted medRxiv a license to display the preprint in perpetuity.
It is made available under a CC-BY 4.0 International license .

Modeling Factors Influencing Introgression

To determine predictors of *w*Mel introgression across release areas, we constructed spatiotemporal regression models using the Integrated Nested Laplace Approximation (INLA) method using the R-INLA package (38). INLA approximates Bayesian inference and addresses the spatial and temporal autocorrelation of variables. All models used the trap-specific extrapolated *w*Mel-positive count at time *t* as the outcome variable, calculated by multiplying the trap prevalence of *w*Mel by the total *Ae. aegypti* caught, modeled with a negative binomial likelihood to account for overdispersion, with an offset of the total *Ae. aegypti* caught at time *t*. The models also included a time-varying autoregressive term of order 1 and a spatially varying autoregressive term using stochastic partial differential equations (**Figures S6 and S13**).

Prior to model-building, we restricted the possible meteorologic, socioeconomic, and environmental covariates included. We examined the correlation between covariates using a Spearman-rank correlation test (**Figure S3**). For highly correlated variables, we fit separate models using each and determined the best fit. For temporally varying covariates, we also examined the correlation between the model outcome (extrapolated *w*Mel-positive count) and the covariate at lags zero to eight weeks in the past using a cross correlation function (**Figure S2**). We only included significant lags in our models and restricted further based on significance. We determined whether to include covariates as linear or non-linear using generalized additive models of the covariate, the model outcome (extrapolated *w*Mel-positive count), and an offset (total *Ae. aegypti* caught) (**Figure S1**). Non-linear variables were included in the model using quintiles, fit with a random walk order of two in INLA.

To assess the robustness of our approach, we performed several sensitivity analyses of our models. We tried using monthly and yearly AR1 temporal autocorrelation terms, to ensure that we were not overfitting our model and taking away the temporally varying effects of our covariates (**Figures S7, S8**). We varied the radii surrounding the trap to 50m and 200m, to ensure that our results were not being driven by the spatial scale to which we aggregated our data (**Figures S9, S10**). We restricted our data to only areas which received releases from vehicles (**Figure S11**). Releases were performed using backpacks in areas with informal settlements and areas with security concerns, where release schedules often needed to be modified. To check that our results were not being driven by variability in releases, we restricted our analysis to areas which received the intervention as planned. We restricted our analyses to a 50% random subsample of space-time observations, to ensure we were not overfitting to our data (**Figure S12**). We restricted our analyses to space-time observations without ongoing *w*Mel releases, to ensure that recapturing recently released *w*Mel *Ae. aegypti* (i.e. contamination) was not biasing our results (**Figure S13**). To determine whether the effects of environmental, socioeconomic, and meteorological factors on introgression were being mediated by *Ae. aegypti* abundance, we fit our model without *Ae. aegypti* trap counts or previous *w*Mel introgression measures (**Figure S4**). Finally, to determine the effect of seasonality separately from the weather covariates, we included a monthly cyclic random walk term (**Figure S5**).

Baseline Abundance Measurement

To better understand the wildtype *w*Mel-negative *Ae. aegypti* population in the study areas and investigate predictors of introgression that could be measured at beginning of releases, we used the initial *w*Mel releases as a Mark-Release-Recapture experiment. Given that *w*Mel begins spreading through a wildtype population due to maternal inheritance and cytoplasmic incompatibility, we only included traps for which the first trap collection occurred 36 days or less after the first release (minimum of 9 days present in the data). This reduces the risk of mistakenly counting introgressed *w*Mel *Ae. aegypti* as newly released *w*Mel *Ae. aegypti*, which would lead to underestimating mosquito abundance in areas with better introgression. We also excluded traps that detected no *w*Mel-positive *Ae. aegypti* in the first trapping event. We included 85 traps in this analysis.

medRxiv preprint doi: https://doi.org/10.64898/2026.10.16.26365711; this version posted October 21, 2026. The copyright holder for this preprint (which was not certified by peer review) is the author/funder, who has granted medRxiv a license to display the preprint in perpetuity.
It is made available under a CC-BY 4.0 International license .

We calculated a previously validated metric, the Fisher-Ford Index, defined as $N_t = Mn\phi^t/m$, where M is the number of *w*Mel *Ae. aegypti* released, n is the total number of *Ae. aegypti* captured the following week, m is the number of *w*Mel *Ae. aegypti* captured the following week, ϕ is the probability of daily survival of a *w*Mel *Ae. aegypti*, and t is the number of days between *w*Mel release and trap collection. We set ϕ to 0.82 based on previous studies (23). We included all mosquito releases in a 100m radius around each trap before the first collection event. We calculated the FFI and the ratio between the average number of mosquitoes released before the first trapping event and the FFI. We tested for associations between both of these metrics and mean introgression using generalized linear regressions. To assess robustness to the assumed ϕ, we repeated these regressions varying ϕ from 0.75-0.90 in increments of 0.01. To ensure that traps were not disproportionately sampling the same population leading to synthetic differences in abundances, we repeated these analyses aggregating traps by 100m cells (**Figure S16**).

To determine the sensitivity and specificity of the mosquitoes released/FFI ratio to predict mean introgression throughout the study period, we plotted a receiver operating characteristic (ROC) curve for a threshold of 20% mean introgression (median introgression for traps for which FFI could be calculated) (**Figure S15**).

All statistical analyses were conducted using R Statistical Software (version 4.4.1, R Foundation for Statistical Computing, Vienna, Austria) (39).

Supplementary figures

medRxiv preprint doi: https://doi.org/10.64898/2026.10.16.26365711; this version posted October 21, 2026. The copyright holder for this preprint (which was not certified by peer review) is the author/funder, who has granted medRxiv a license to display the preprint in perpetuity.
It is made available under a CC-BY 4.0 International license .

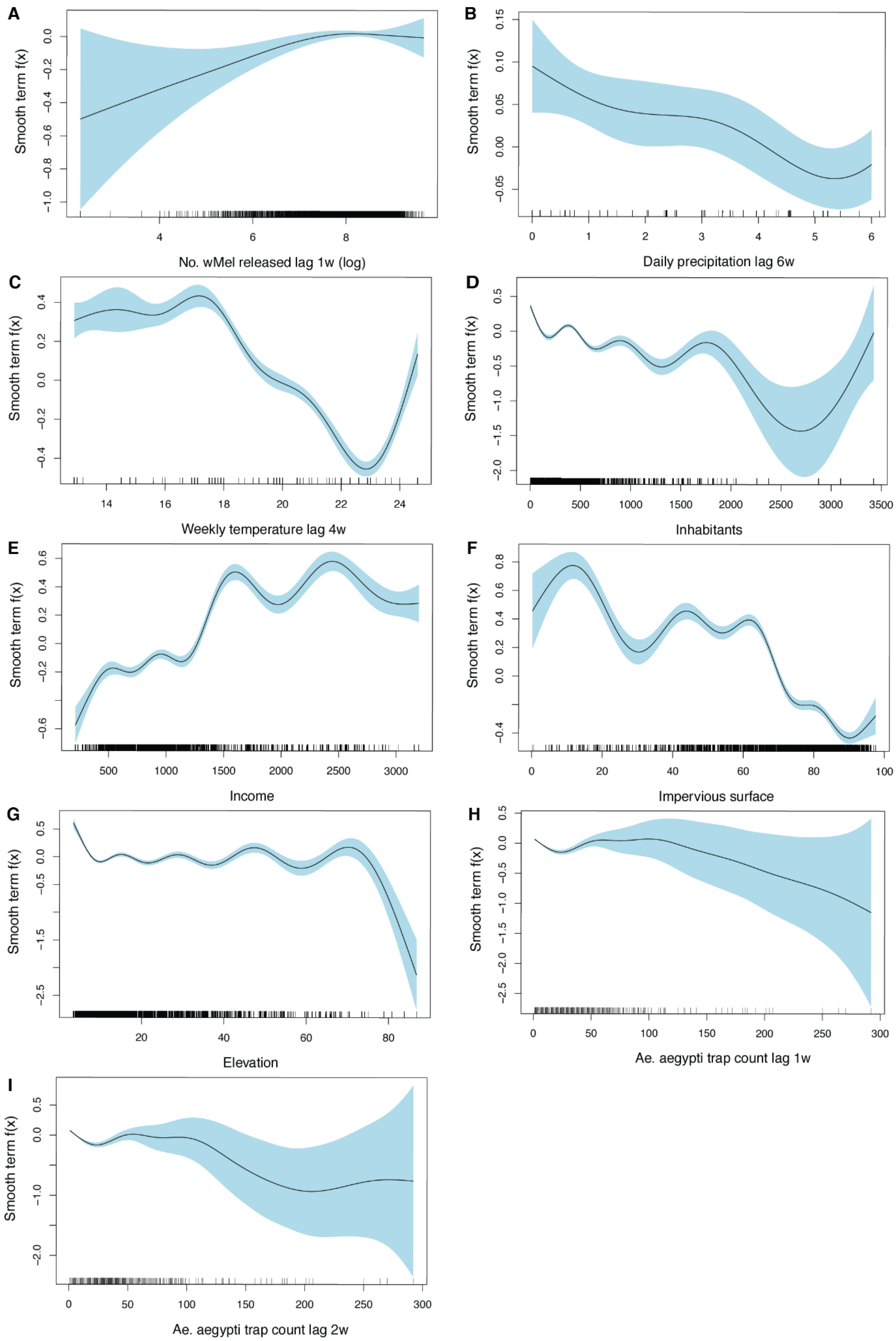

Figure S1. Generalized additive models used to determine covariate linearity for spatiotemporal INLA models. Univariate generalized additive models (GAMs) were run on each variable prior to

medRxiv preprint doi: https://doi.org/10.64898/2026.10.16.26365711; this version posted October 21, 2026. The copyright holder for this preprint (which was not certified by peer review) is the author/funder, who has granted medRxiv a license to display the preprint in perpetuity.
It is made available under a CC-BY 4.0 International license .

inclusion in the spatiotemporal INLA models, to determine the linear of their relationship with the outcome variable of extrapolated *w*Mel count (prevalence*total *Ae. aegypti* caught by the trap). The results of the variables included in the final models are shown above. All GAMs included the outcome of extrapolated *w*Mel count, the variable of interest shown on the x-axis, and an offset of total *Ae. aegypti* trap count through time at the trap-level. The association found is shown by the black line, surrounded by a 95% confidence interval. Along the x-axis, there is a rug plot describing the distribution of the data.

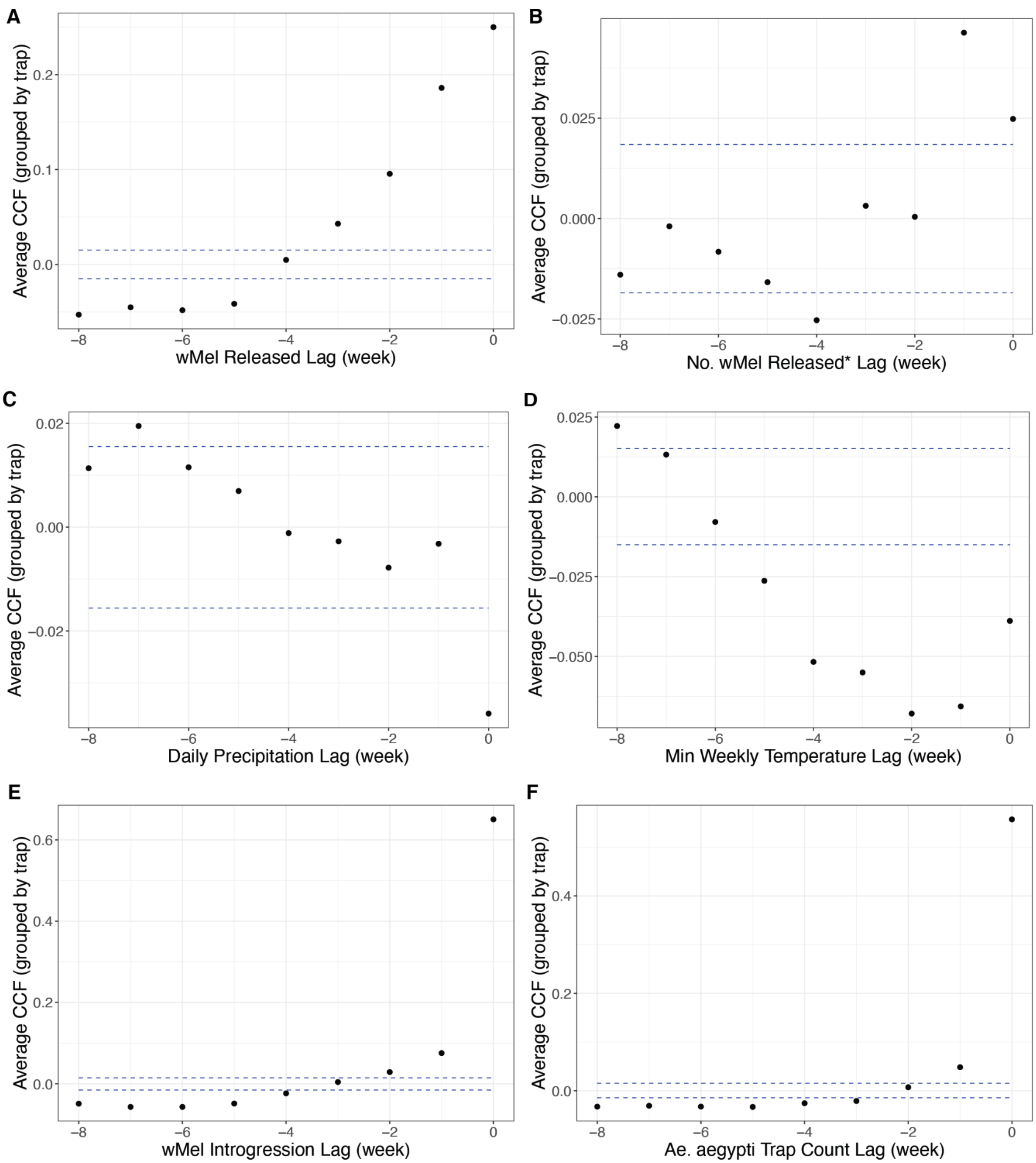

Figure S2. Cross correlation function results used to determine significant temporal lags of covariates to be tested in spatiotemporal INLA models. The correlation of all temporally-varying

medRxiv preprint doi: https://doi.org/10.64898/2026.10.16.26365711; this version posted October 21, 2026. The copyright holder for this preprint (which was not certified by peer review) is the author/funder, who has granted medRxiv a license to display the preprint in perpetuity.
It is made available under a CC-BY 4.0 International license .

variables were tested using historical lags 0-8 weeks against the outcome variable of extrapolated *w*Mel count (prevalence*total *Ae. aegypti* caught by the trap) for each trap. The results of the variables included in the final models are shown above. The mean correlation coefficient for all traps is shown for each lag by the black dots. The blue dashed lines indicate the 95% thresholds of significance. Only lags with coefficients outside of the threshold lines prior to lags becoming nonsignificant were included in the models.

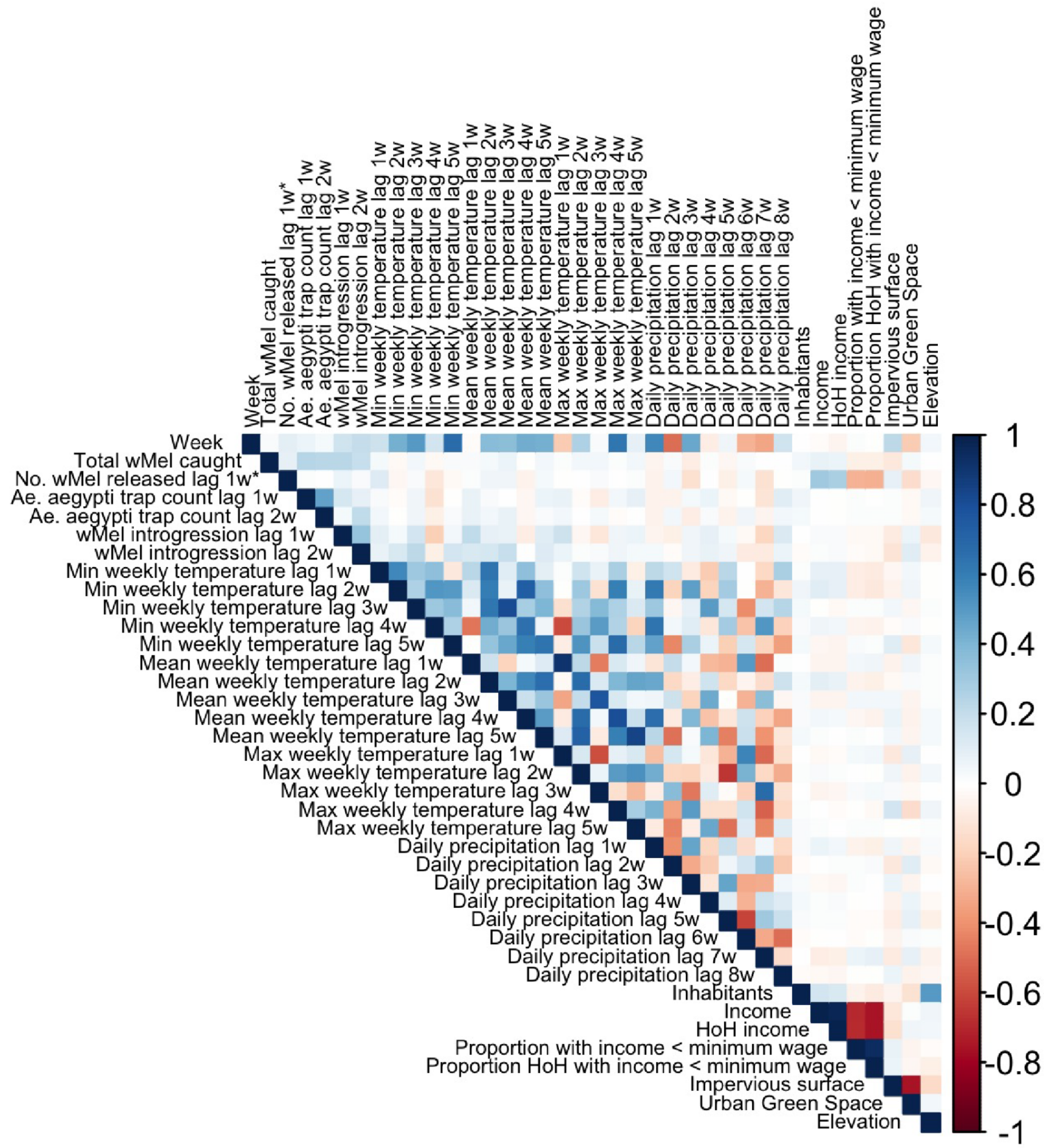

Figure S3. Correlation of covariates tested in spatiotemporal models. Correlation was estimated using a Spearman-rank correlation test arranged by week date and grouped by trap. Darker blue cells indicate high positive temporal correlation. White cells (0) indicate no temporal correlation. Darker red cells indicate high negative temporal correlation. *w*Mel released lag 1w could not be included due to its binary structure.

medRxiv preprint doi: https://doi.org/10.64898/2026.10.16.26365711; this version posted October 21, 2026. The copyright holder for this preprint (which was not certified by peer review) is the author/funder, who has granted medRxiv a license to display the preprint in perpetuity.
It is made available under a CC-BY 4.0 International license .

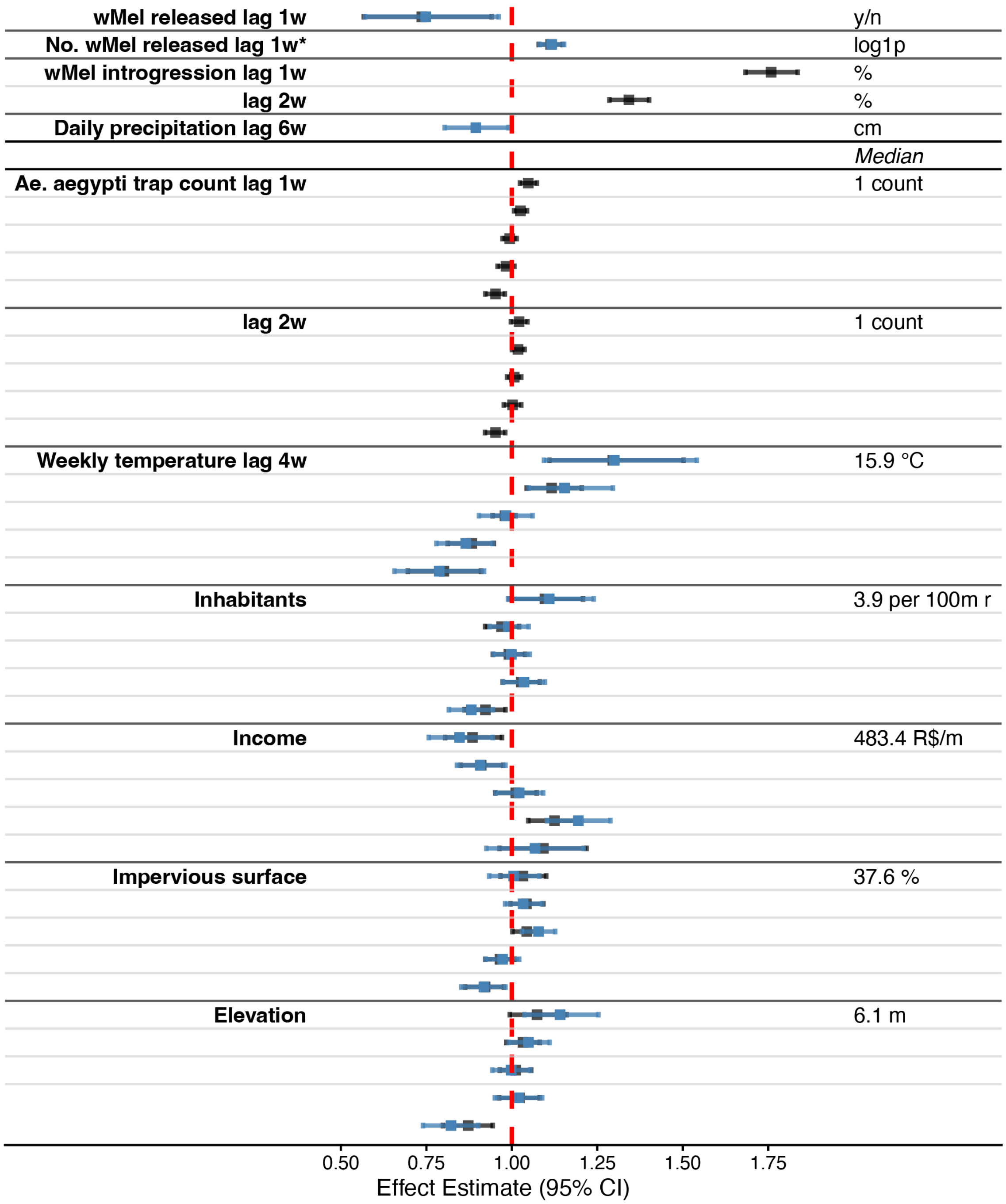

Figure S4. **Spatiotemporal analysis of environmental, SE and meteorological factors associated without *w*Mel introgression** (Conditional R^2=0.75). The effects of the original model are shown in black, while the sensitivity analysis model effects are shown in blue. The outcome of the model is extrapolated *w*Mel count (*w*Mel prevalence**Ae. aegypti* caught). Non-linear effects were run as quintiles, depicted in the distribution plots, with red lines marking the boundaries, and medians and units of measurement in the right text column. All data was aggregated by week and scaled to the 100m radius surrounding traps. '*w*Mel released lag 1w' is a binary term, indicating whether releases occurred; 'No. *w*Mel released lag 1w*' is the log(1+x) or log1p number of mosquitoes released; 'Daily precipitation lag 6w' is the mean daily precipitation, in centimeters; 'Weekly temperature lag 4w' is the minimum weekly

medRxiv preprint doi: https://doi.org/10.64898/2026.10.16.26365711; this version posted October 21, 2026. The copyright holder for this preprint (which was not certified by peer review) is the author/funder, who has granted medRxiv a license to display the preprint in perpetuity.
It is made available under a CC-BY 4.0 International license .

temperature, in degrees Celsius; ‘Inhabitants’ is the population density; ‘Income’ is the mean income in R$ for persons over 10; ‘Impervious surface’ is the percent area comprised of water-impervious surfaces; ‘Elevation’ is measured in meters.

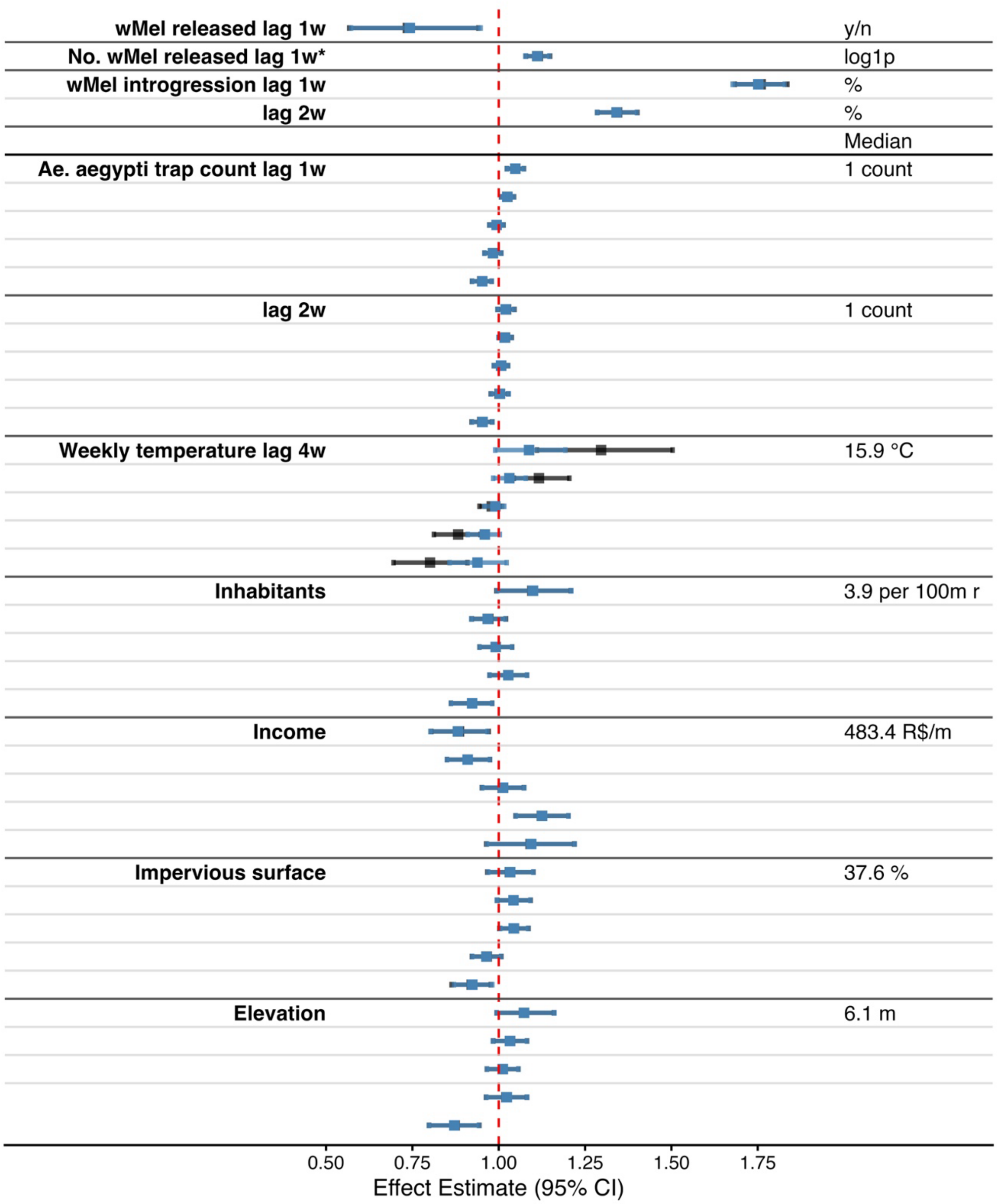

Figure S5. Model with monthly cyclic random walk term for seasonality: Spatiotemporal analysis of factors measured during the intervention associated with *w*Mel introgression. The effects of the original

medRxiv preprint doi: https://doi.org/10.64898/2026.10.16.26365711; this version posted October 21, 2026. The copyright holder for this preprint (which was not certified by peer review) is the author/funder, who has granted medRxiv a license to display the preprint in perpetuity.
It is made available under a CC-BY 4.0 International license .

model are shown in black, while the sensitivity analysis model effects are shown in blue. Several lags of variables only show effects from the sensitivity analysis model, as they were not significant in the main model. The outcome is extrapolated *w*Mel count (*w*Mel prevalence**Ae. aegypti* caught). Terms above *Median* were included as linear, and below as non-linear. Non-linear effects were run as quintiles, depicted in the distribution plots, with red lines marking the boundaries, and medians and units of measurement in the text column. Data was aggregated by week and scaled to the 100m radius surrounding traps. '*w*Mel released lag 1w' is a binary term, indicating whether releases occurred; 'No. *w*Mel released lag 1w*' is the log(1+x) number of mosquitoes released; '*w*Mel introgression lag 1w, 2w' is the percent *w*Mel-positive mosquitoes; 'Daily precipitation lag 6w' is the mean daily precipitation, in centimeters; '*Ae. aegypti* trap count lag 1w, 2w' are measures of *Ae. aegypti* abundance, distribution tails not depicted; 'Weekly temperature lag 4w' is the minimum weekly temperature, in Celsius; 'Inhabitants' is the population density; 'HoH income' is the mean head of household income, but the main model used the original 'Income' variable; 'Impervious surface' is the percent area comprised of water-impervious surfaces; 'Elevation' is in meters.

medRxiv preprint doi: https://doi.org/10.64898/2026.10.16.26365711; this version posted October 21, 2026. The copyright holder for this preprint (which was not certified by peer review) is the author/funder, who has granted medRxiv a license to display the preprint in perpetuity.
It is made available under a CC-BY 4.0 International license .

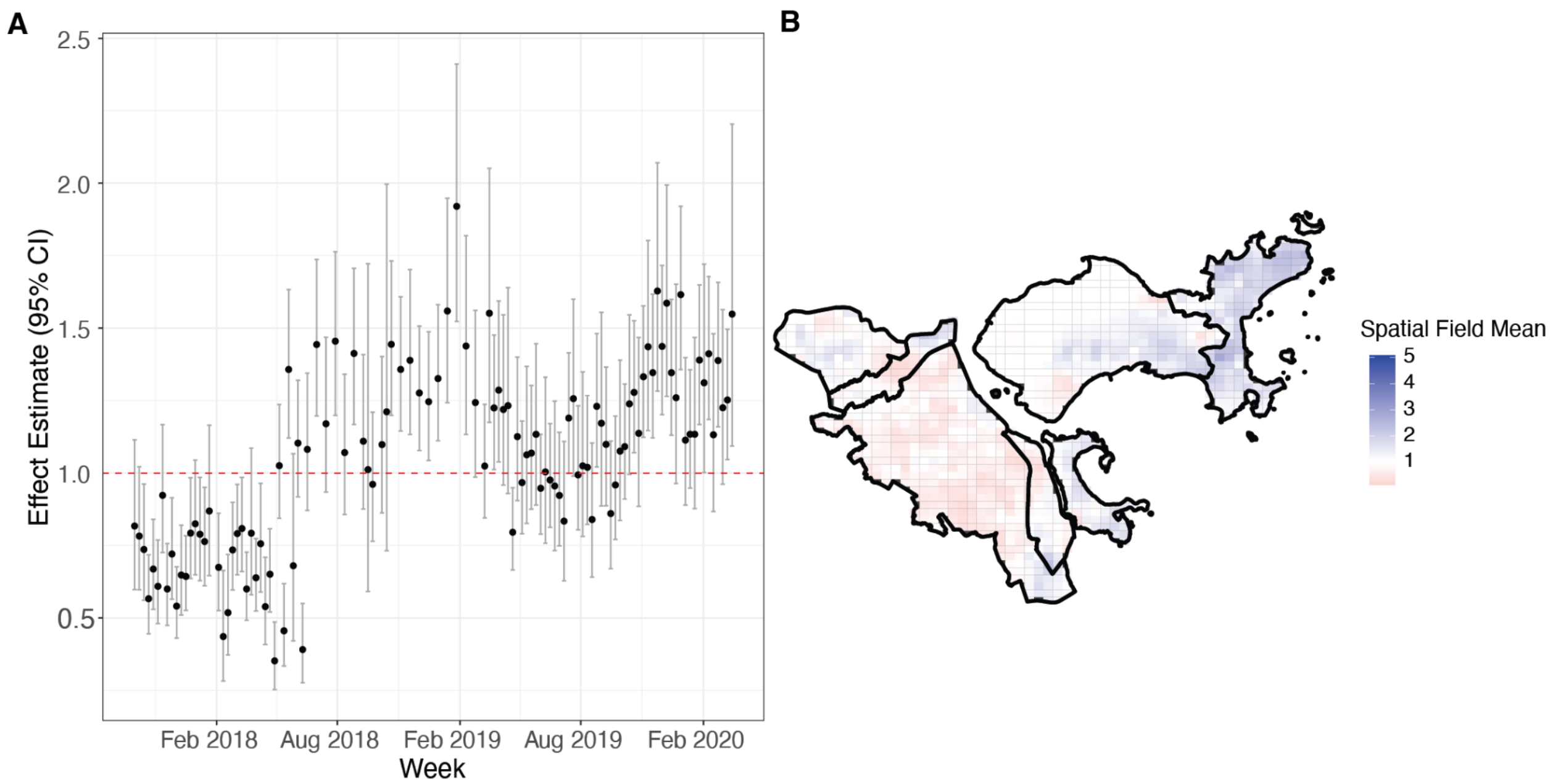

Figure S6. Model temporal and spatial autoregressive terms. A) The temporal autoregressive term was fit using a weekly autoregressive order one (AR1) term in INLA. The weekly autoregressive effect fit is shown by the black dots**,** with corresponding 95% confidence intervals in gray. The effects show slight seasonality, as evidenced by the slight peaks and troughs in effects. There is also a switch from negative to positive around week 40, which corresponds to the onset of consolidation releases in the RJ3 areas. This is likely due to the frequency-dependent fitness effects of *w*Mel introgression (cytoplasmic incompatibility and maternal inheritance). B) The spatial autoregressive term was fit using stochastic partial differentiation equations (SPDE) fit to a mesh of trap-level geolocations. The spatial field mean shows the average spatial variation across the study area through time. Areas with lower spatial field means, shown in red, likely have a weaker underlying spatial process. Whereas areas with higher spatial field means, shown in blue, have a stronger underlying spatial process and may correspond to hotspots of high introgression.

medRxiv preprint doi: https://doi.org/10.64898/2026.10.16.26365711; this version posted October 21, 2026. The copyright holder for this preprint (which was not certified by peer review) is the author/funder, who has granted medRxiv a license to display the preprint in perpetuity.
It is made available under a CC-BY 4.0 International license .

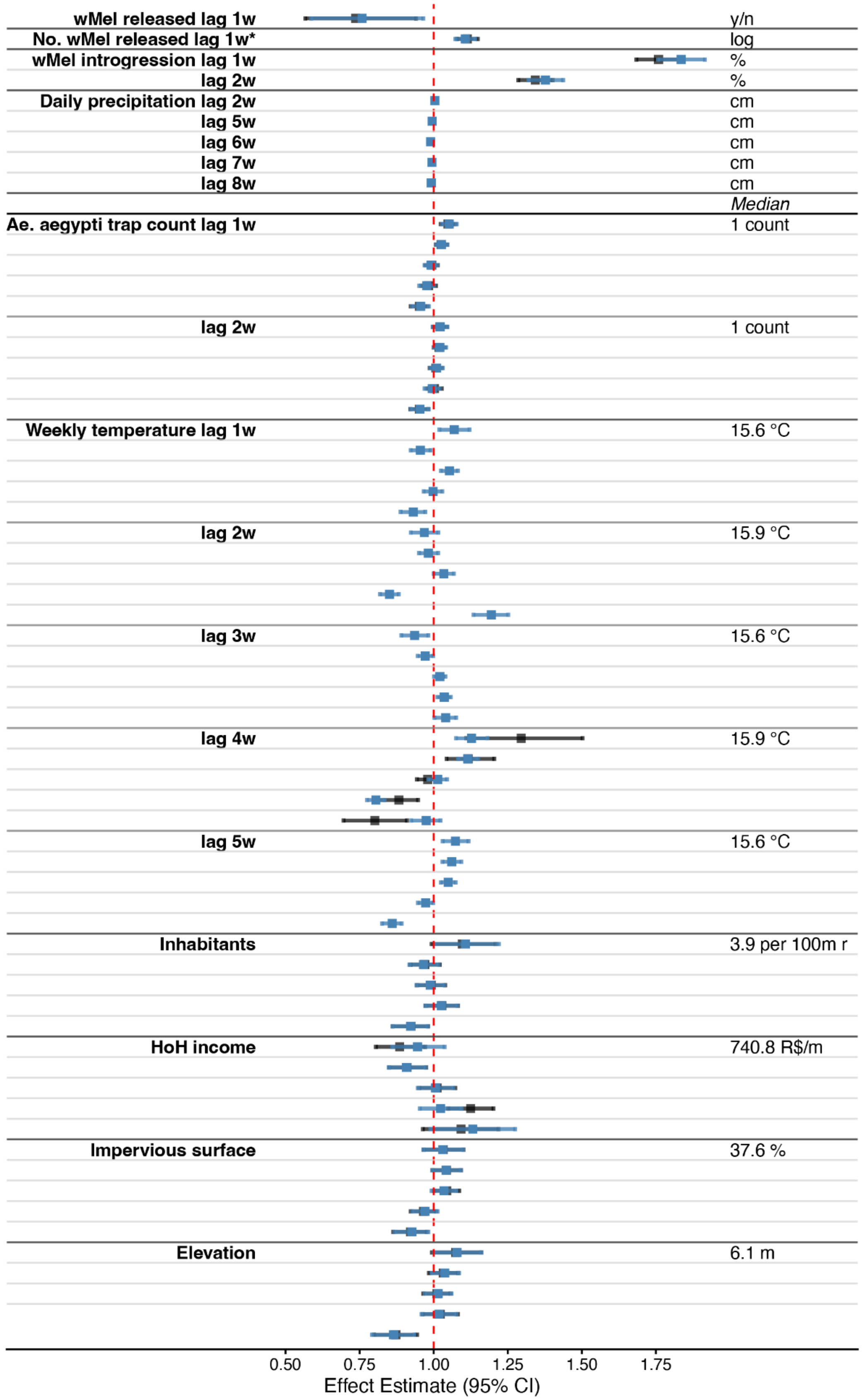

695

medRxiv preprint doi: https://doi.org/10.64898/2026.10.16.26365711; this version posted October 21, 2026. The copyright holder for this preprint (which was not certified by peer review) is the author/funder, who has granted medRxiv a license to display the preprint in perpetuity.
It is made available under a CC-BY 4.0 International license .

Figure S7. Model with monthly AR1 term: Spatiotemporal analysis of factors measured during the intervention associated with *w*Mel introgression. The effects of the original model are shown in black, while the sensitivity analysis model effects are shown in blue. Several lags of variables only show effects from the sensitivity analysis model, as they were not significant in the main model. The outcome is extrapolated *w*Mel count (*w*Mel prevalence**Ae. aegypti* caught). Terms above *Median* were included as linear, and below as non-linear. Non-linear effects were run as quintiles, depicted in the distribution plots, with red lines marking the boundaries, and medians and units of measurement in the text column. Data was aggregated by week and scaled to the 100m radius surrounding traps. '*w*Mel released lag 1w' is a binary term, indicating whether releases occurred; 'No. *w*Mel released lag 1w*' is the log(1+x) number of mosquitoes released; '*w*Mel introgression lag 1w, 2w' is the percent *w*Mel-positive mosquitoes; 'Daily precipitation lag 6w' is the mean daily precipitation, in centimeters; '*Ae. aegypti* trap count lag 1w, 2w' are measures of *Ae. aegypti* abundance, distribution tails not depicted; 'Weekly temperature lag 4w' is the minimum weekly temperature, in Celsius; 'Inhabitants' is the population density; 'HoH income' is the mean head of household income, but the main model used the original 'Income' variable; 'Impervious surface' is the percent area comprised of water-impervious surfaces; 'Elevation' is in meters.

medRxiv preprint doi: https://doi.org/10.64898/2026.10.16.26365711; this version posted October 21, 2026. The copyright holder for this preprint (which was not certified by peer review) is the author/funder, who has granted medRxiv a license to display the preprint in perpetuity.
It is made available under a CC-BY 4.0 International license .

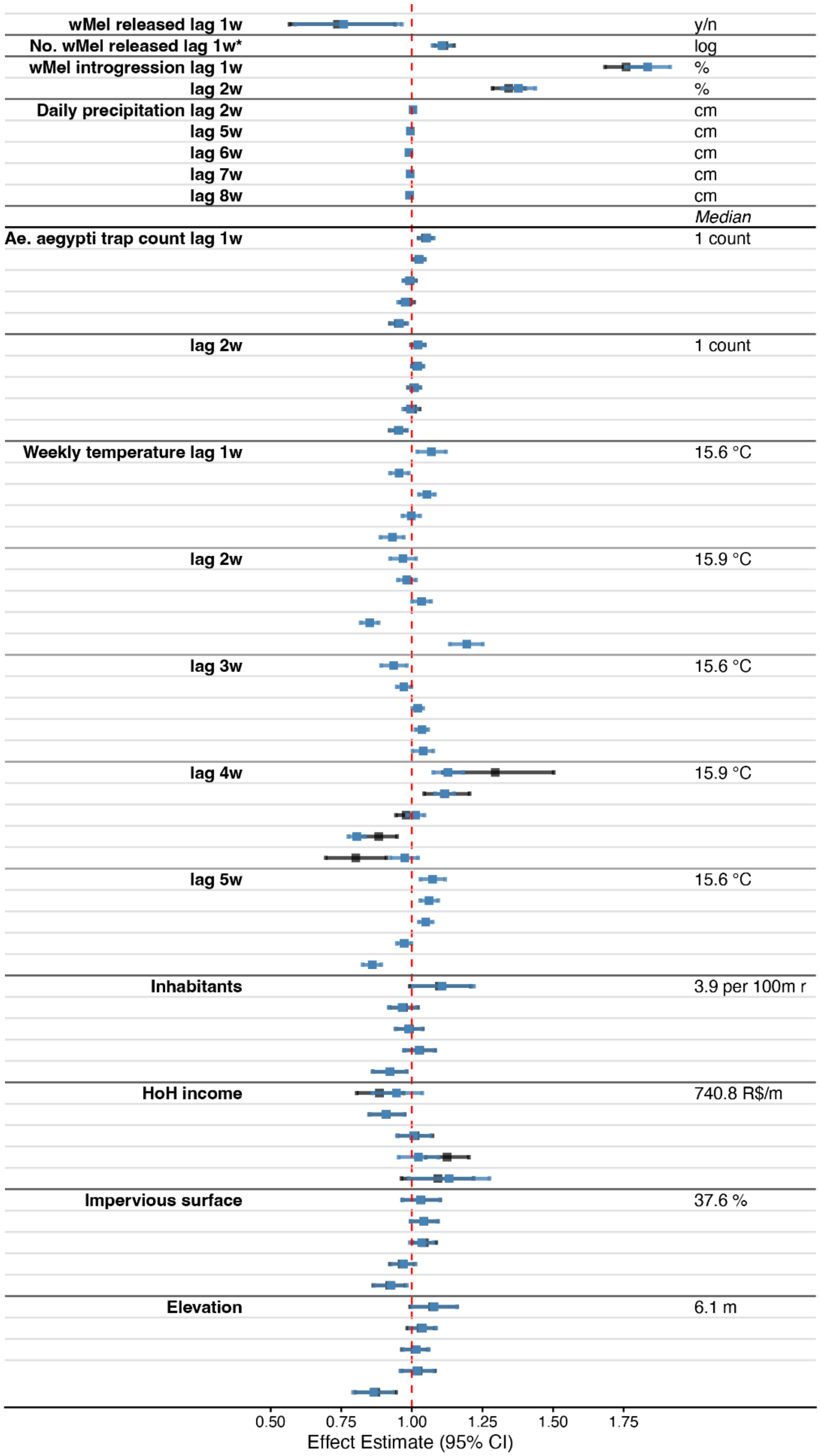

714

medRxiv preprint doi: https://doi.org/10.64898/2026.10.16.26365711; this version posted October 21, 2026. The copyright holder for this preprint (which was not certified by peer review) is the author/funder, who has granted medRxiv a license to display the preprint in perpetuity.
It is made available under a CC-BY 4.0 International license .

Figure S8. Model with yearly AR1 term: Spatiotemporal analysis of factors measured during the intervention associated with *w*Mel introgression. The effects of the original model are shown in black, while the sensitivity analysis model effects are shown in blue. Several lags of variables only show effects from the sensitivity analysis model, as they were not significant in the main model. The outcome is extrapolated *w*Mel count (*w*Mel prevalence**Ae. aegypti* caught). Terms above *Median* were included as linear, and below as non-linear. Non-linear effects were run as quintiles, depicted in the distribution plots, with red lines marking the boundaries, and medians and units of measurement in the text column. Data was aggregated by week and scaled to the 100m radius surrounding traps. '*w*Mel released lag 1w' is a binary term, indicating whether releases occurred; 'No. *w*Mel released lag 1w*' is the log(1+x) number of mosquitoes released; '*w*Mel introgression lag 1w, 2w' is the percent *w*Mel-positive mosquitoes; 'Daily precipitation lag 6w' is the mean daily precipitation, in centimeters; '*Ae. aegypti* trap count lag 1w, 2w' are measures of *Ae. aegypti* abundance, distribution tails not depicted; 'Weekly temperature lag 4w' is the minimum weekly temperature, in Celsius; 'Inhabitants' is the population density; 'HoH income' is the mean head of household income, but the main model used the original 'Income' variable; 'Impervious surface' is the percent area comprised of water-impervious surfaces; 'Elevation' is in meters.

medRxiv preprint doi: https://doi.org/10.64898/2026.10.16.26365711; this version posted October 21, 2026. The copyright holder for this preprint (which was not certified by peer review) is the author/funder, who has granted medRxiv a license to display the preprint in perpetuity.
It is made available under a CC-BY 4.0 International license .

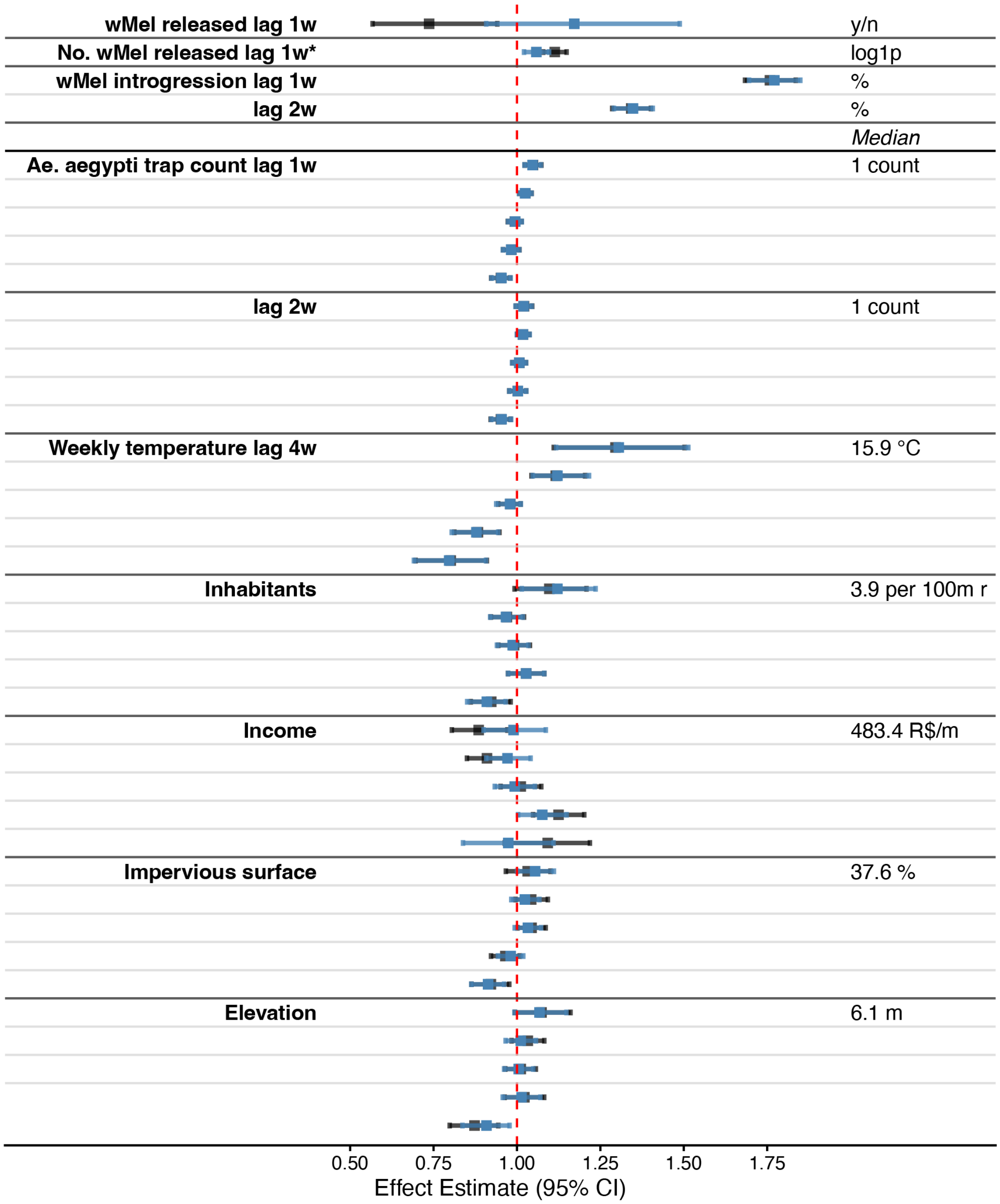

Figure S9. Model using covariates from a 50m radius surrounding traps: Spatiotemporal analysis of factors measured during the intervention associated with *w*Mel introgression. The effects of the original model are shown in black, while the sensitivity analysis model effects are shown in blue. The outcome is extrapolated *w*Mel count (*w*Mel prevalence**Ae. aegypti* caught). Terms above *Median* were included as linear, and below as non-linear. Non-linear effects were run as quintiles, depicted in the distribution plots, with red lines marking the boundaries, and medians and units of measurement in the text column. Data was aggregated by week and scaled to the 50m radius surrounding traps. ‘*w*Mel released lag 1w’ is a binary term, indicating whether releases occurred; ‘No. *w*Mel released lag 1w*’ is the log(1+x) number of mosquitoes released; ‘*w*Mel introgression lag 1w, 2w’ is the percent *w*Mel-positive mosquitoes; ‘*Ae.*

medRxiv preprint doi: https://doi.org/10.64898/2026.10.16.26365711; this version posted October 21, 2026. The copyright holder for this preprint (which was not certified by peer review) is the author/funder, who has granted medRxiv a license to display the preprint in perpetuity.
It is made available under a CC-BY 4.0 International license .

aegypti trap count lag 1w, 2w' are measures of *Ae. aegypti* abundance, distribution tails not depicted; 'Weekly temperature lag 4w' is the minimum weekly temperature, in Celsius; 'Inhabitants' is the population density; 'Income' is the mean income in R$ for persons over 10; 'Impervious surface' is the percent area comprised of water-impervious surfaces; 'Elevation' is in meters.

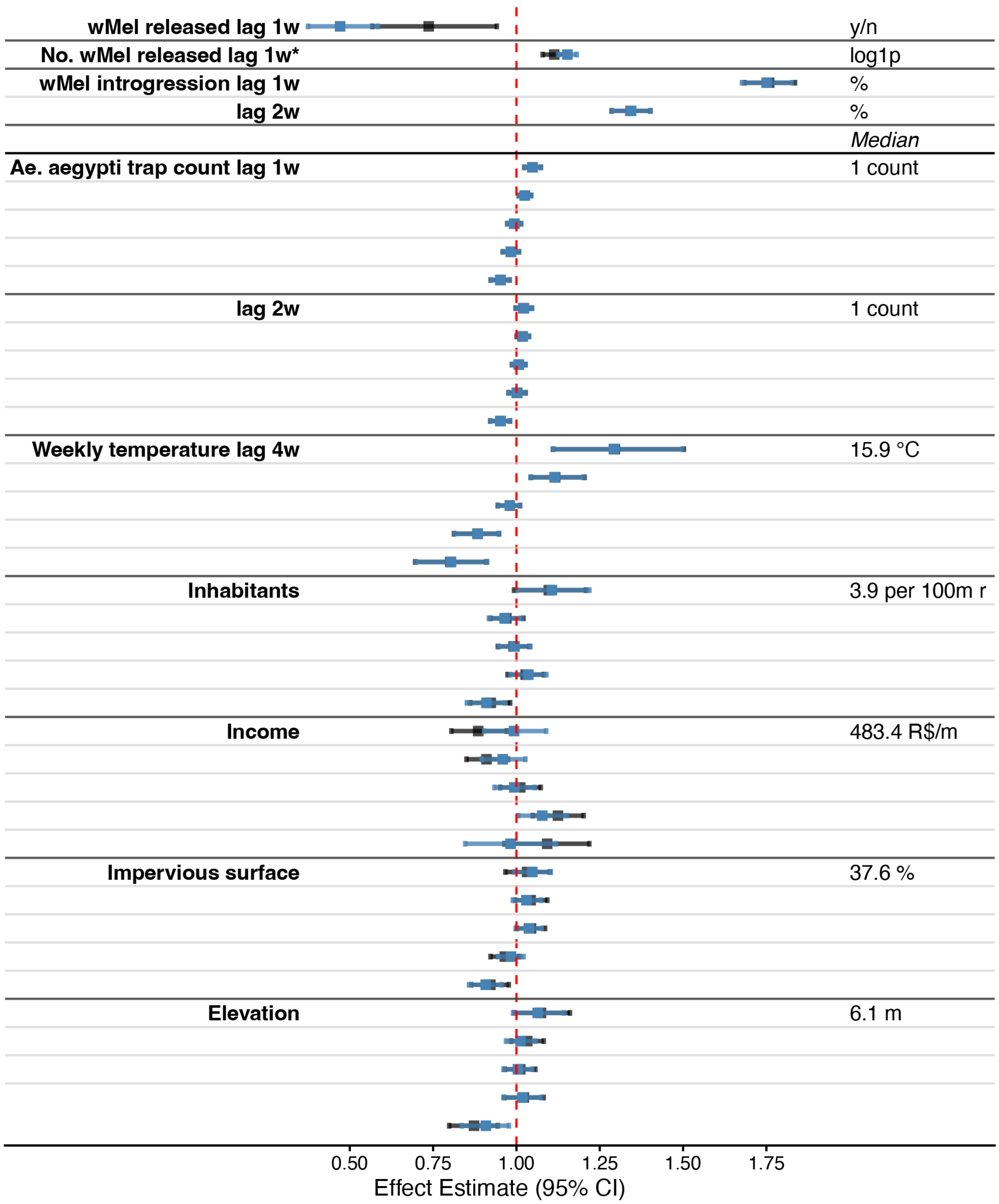

Figure S10. Model using covariates from a 200m radius surrounding traps: Spatiotemporal analysis of factors measured during the intervention associated with *w*Mel introgression. The effects of the original model are shown in black, while the sensitivity analysis model effects are shown in blue. The outcome is extrapolated *w*Mel count (*w*Mel prevalence**Ae. aegypti* caught). Terms above *Median* were

medRxiv preprint doi: https://doi.org/10.64898/2026.10.16.26365711; this version posted October 21, 2026. The copyright holder for this preprint (which was not certified by peer review) is the author/funder, who has granted medRxiv a license to display the preprint in perpetuity.
It is made available under a CC-BY 4.0 International license .

included as linear, and below as non-linear. Non-linear effects were run as quintiles, depicted in the distribution plots, with red lines marking the boundaries, and medians and units of measurement in the text column. Data was aggregated by week and scaled to the 200m radius surrounding traps. '*w*Mel released lag 1w' is a binary term, indicating whether releases occurred; 'No. *w*Mel released lag 1w*' is the log(1+x) number of mosquitoes released; '*w*Mel introgression lag 1w, 2w' is the percent *w*Mel-positive mosquitoes; '*Ae. aegypti* trap count lag 1w, 2w' are measures of *Ae. aegypti* abundance, distribution tails not depicted; 'Weekly temperature lag 4w' is the minimum weekly temperature, in Celsius; 'Inhabitants' is the population density; 'Income' is the mean income in R$ for persons over 10; 'Impervious surface' is the percent area comprised of water-impervious surfaces; 'Elevation' is in meters.

medRxiv preprint doi: https://doi.org/10.64898/2026.10.16.26365711; this version posted October 21, 2026. The copyright holder for this preprint (which was not certified by peer review) is the author/funder, who has granted medRxiv a license to display the preprint in perpetuity.
It is made available under a CC-BY 4.0 International license .

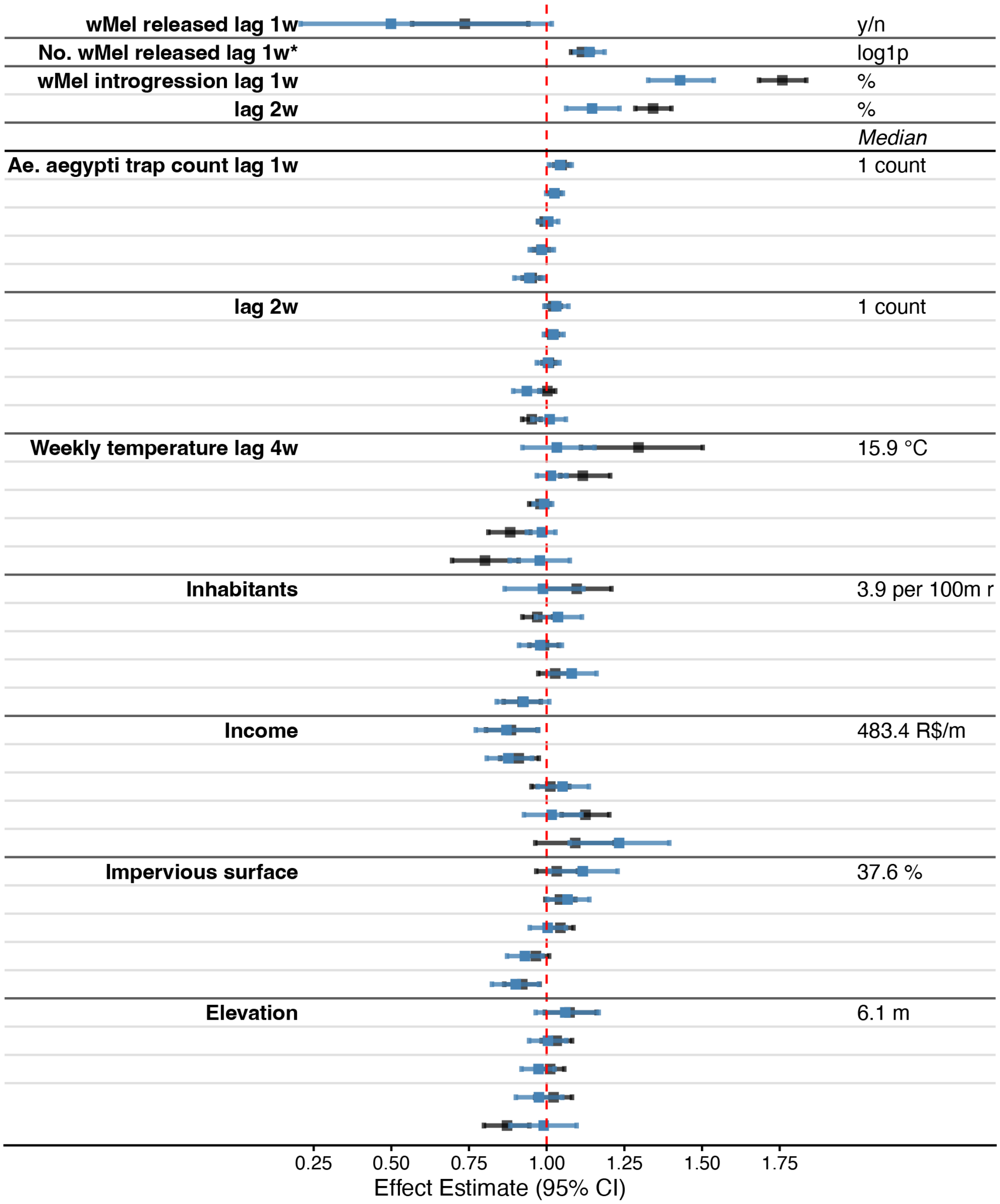

Figure S11. Model using only space-time observations from areas with vehicle-based *w*Mel releases: Spatiotemporal analysis of factors measured during the intervention associated with *w*Mel introgression. The effects of the original model are shown in black, while the sensitivity analysis model effects are shown in blue. The outcome is extrapolated *w*Mel count (*w*Mel prevalence**Ae. aegypti* caught). Terms above *Median* were included as linear, and below as non-linear. Non-linear effects were run as quintiles, depicted in the distribution plots, with red lines marking the boundaries, and medians and units of measurement in the text column. Data was aggregated by week and scaled to the 100m radius surrounding traps. '*w*Mel released lag 1w' is a binary term, indicating whether releases occurred; 'No. *w*Mel released lag 1w*' is the log(1+x) number of mosquitoes released; '*w*Mel introgression lag 1w, 2w' is the percent

medRxiv preprint doi: https://doi.org/10.64898/2026.10.16.26365711; this version posted October 21, 2026. The copyright holder for this preprint (which was not certified by peer review) is the author/funder, who has granted medRxiv a license to display the preprint in perpetuity.
It is made available under a CC-BY 4.0 International license .

*w*Mel-positive mosquitoes; '*Ae. aegypti* trap count lag 1w, 2w' are measures of *Ae. aegypti* abundance, distribution tails not depicted; 'Weekly temperature lag 4w' is the minimum weekly temperature, in Celsius; 'Inhabitants' is the population density; 'Income' is the mean income in R$ for persons over 10; 'Impervious surface' is the percent area comprised of water-impervious surfaces; 'Elevation' is in meters.

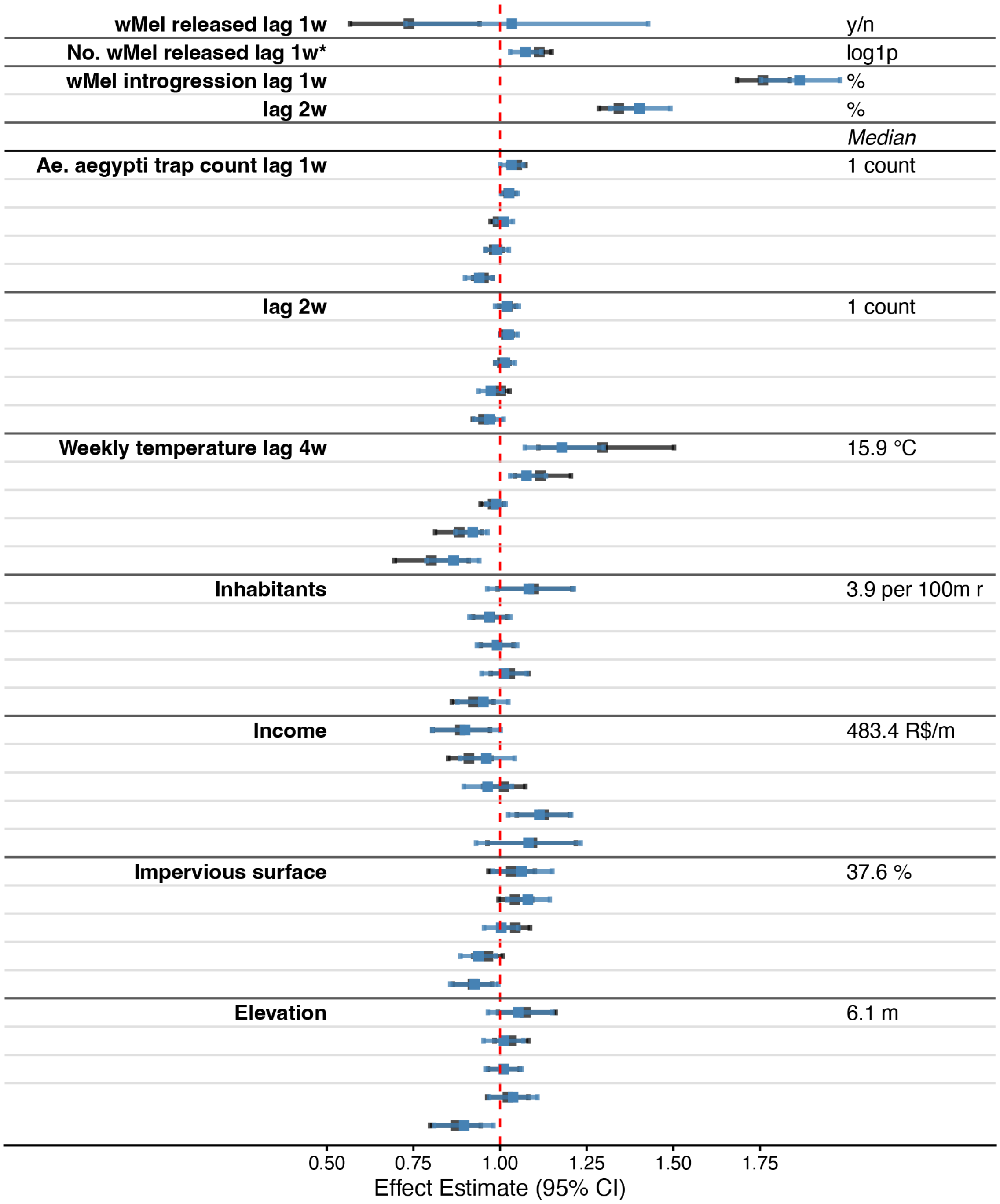

Figure S12. Model using a random 50% subsample of space-time observations: Spatiotemporal analysis of factors measured during the intervention associated with *w*Mel introgression. The effects of the original model are shown in black, while the sensitivity analysis model effects are shown in blue. The outcome is extrapolated *w*Mel count (*w*Mel prevalence**Ae. aegypti* caught). Terms above *Median* were

medRxiv preprint doi: https://doi.org/10.64898/2026.10.16.26365711; this version posted October 21, 2026. The copyright holder for this preprint (which was not certified by peer review) is the author/funder, who has granted medRxiv a license to display the preprint in perpetuity.
It is made available under a CC-BY 4.0 International license .

included as linear, and below as non-linear. Non-linear effects were run as quintiles, depicted in the distribution plots, with red lines marking the boundaries, and medians and units of measurement in the text column. Data was aggregated by week and scaled to the 100m radius surrounding traps. '*w*Mel released lag 1w' is a binary term, indicating whether releases occurred; 'No. *w*Mel released lag 1w*' is the log number of mosquitoes released; '*w*Mel introgression lag 1w, 2w' is the percent *w*Mel-positive mosquitoes; '*Ae. aegypti* trap count lag 1w, 2w' are measures of *Ae. aegypti* abundance, distribution tails not depicted; 'Weekly temperature lag 4w' is the minimum weekly temperature, in Celsius; 'Inhabitants' is the population density; 'Income' is the mean income in R$ for persons over 10; 'Impervious surface' is the percent area comprised of water-impervious surfaces; 'Elevation' is in meters.

medRxiv preprint doi: https://doi.org/10.64898/2026.10.16.26365711; this version posted October 21, 2026. The copyright holder for this preprint (which was not certified by peer review) is the author/funder, who has granted medRxiv a license to display the preprint in perpetuity.
It is made available under a CC-BY 4.0 International license .

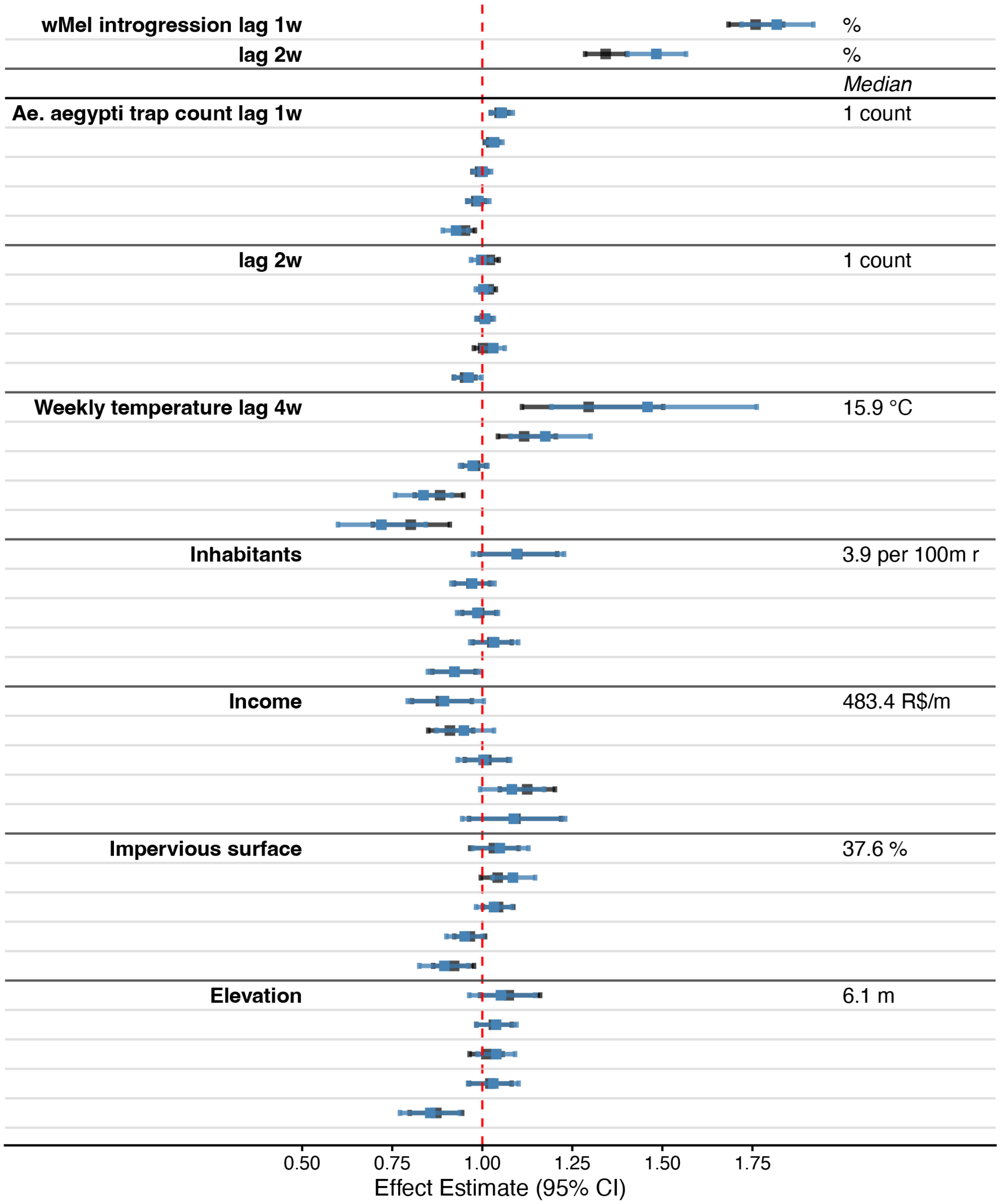

Figure S13. Model using only space-time observations from non-release periods: Spatiotemporal analysis of factors measured during the intervention associated with *w*Mel introgression. The effects of the original model are shown in black, while the sensitivity analysis model effects are shown in blue. The outcome is extrapolated *w*Mel count (*w*Mel prevalence**Ae. aegypti* caught). Terms above *Median* were included as linear, and below as non-linear. Non-linear effects were run as quintiles, depicted in the distribution plots, with red lines marking the boundaries, and medians and units of measurement in the text column. Data was aggregated by week and scaled to the 100m radius surrounding traps. '*w*Mel introgression lag 1w, 2w' is the percent *w*Mel-positive mosquitoes; '*Ae. aegypti* trap count lag 1w, 2w' are measures of *Ae. aegypti* abundance, distribution tails not depicted; 'Weekly temperature lag 4w' is the

medRxiv preprint doi: https://doi.org/10.64898/2026.10.16.26365711; this version posted October 21, 2026. The copyright holder for this preprint (which was not certified by peer review) is the author/funder, who has granted medRxiv a license to display the preprint in perpetuity.
It is made available under a CC-BY 4.0 International license .

minimum weekly temperature, in Celsius; ‘Inhabitants’ is the population density; ‘Income’ is the mean income in R$ for persons over 10; ‘Impervious surface’ is the percent area comprised of water-impervious surfaces; ‘Elevation’ is in meters.

medRxiv preprint doi: https://doi.org/10.64898/2026.10.16.26365711; this version posted October 21, 2026. The copyright holder for this preprint (which was not certified by peer review) is the author/funder, who has granted medRxiv a license to display the preprint in perpetuity.
It is made available under a CC-BY 4.0 International license .

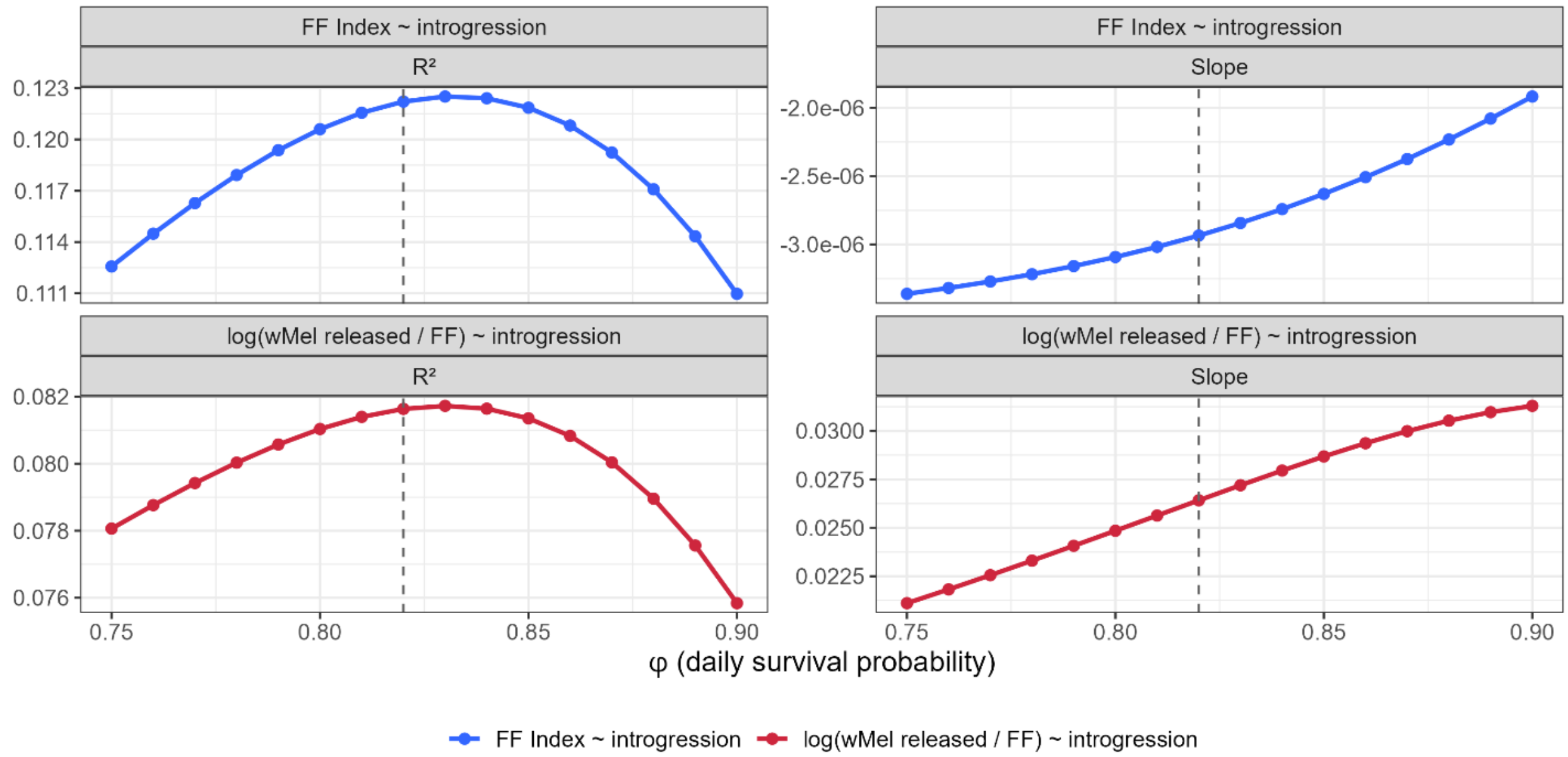

807
808 **Figure S14**. **Sensitivity of associations between Fisher-Ford Index and *w*Mel introgression to daily**
809 **survival probability assumptions**. Facets on the left show explained deviance (R^2) and facets on the
810 right show slope for linear regression of Fisher-Ford (FF) Index and ratio of *w*Mel released (mean of
811 releases before first trapping event) to Fisher-Ford Index versus mean *w*Mel introgression (log-
812 transformed).

medRxiv preprint doi: https://doi.org/10.64898/2026.10.16.26365711; this version posted October 21, 2026. The copyright holder for this preprint (which was not certified by peer review) is the author/funder, who has granted medRxiv a license to display the preprint in perpetuity.
It is made available under a CC-BY 4.0 International license .

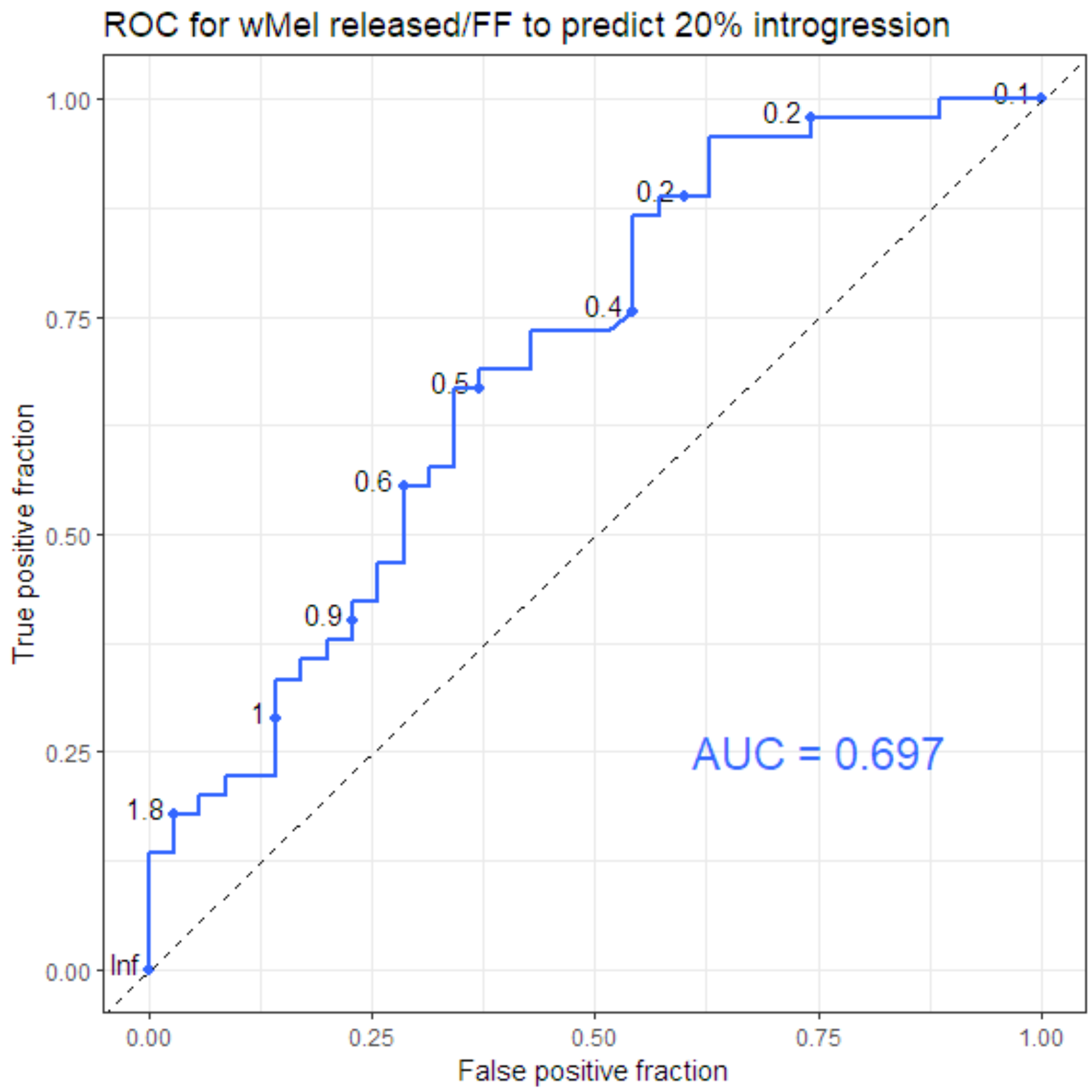

Figure S15. **Receiver operating characteristic (ROC) curve** showing classifying performance of the *w*Mel released/Fisher Ford Index to predict mean introgression at a 20% threshold.

medRxiv preprint doi: https://doi.org/10.64898/2026.10.16.26365711; this version posted October 21, 2026. The copyright holder for this preprint (which was not certified by peer review) is the author/funder, who has granted medRxiv a license to display the preprint in perpetuity.
It is made available under a CC-BY 4.0 International license .

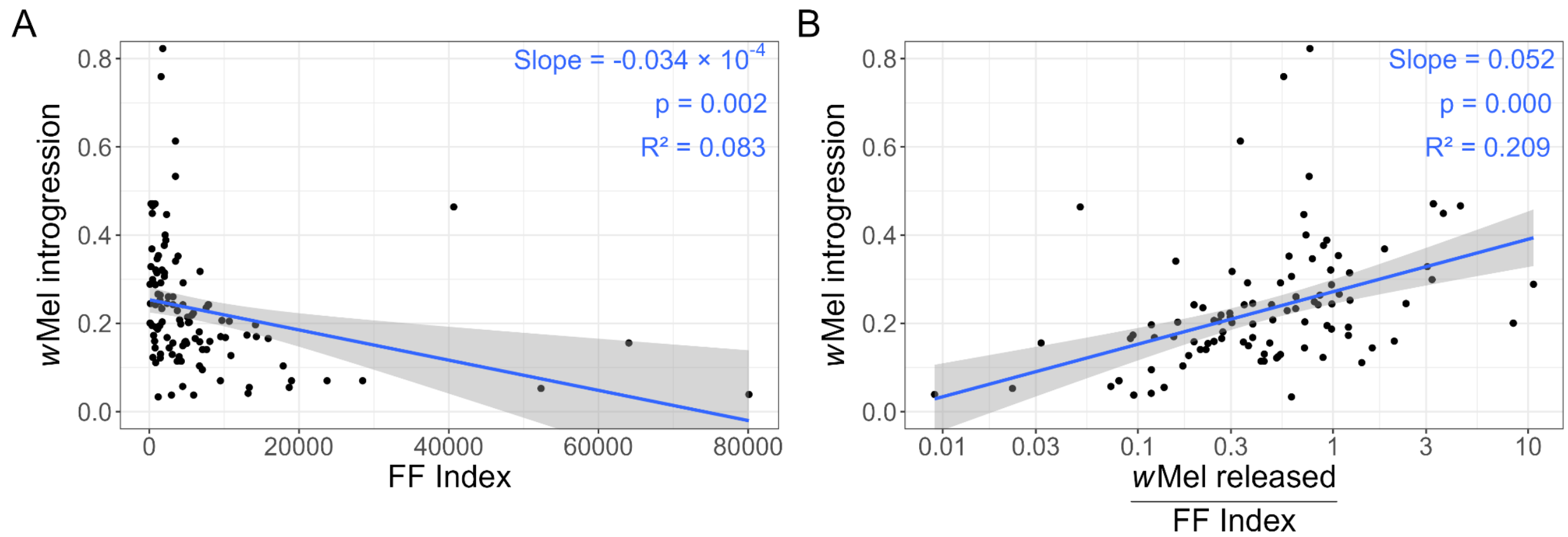

Figure S16. Baseline abundance estimates and introgression in traps with 9-36 days between first release and first trapping event, aggregated by 100m cells. Panels A and B show Fisher-Ford (FF) Index and ratio of wMel released (mean of releases before first trapping event) to Fisher-Ford Index versus mean *w*Mel introgression (log-transformed) for 117 cells. Blue lines show the linear regression trend with 95% CI. Blue text indicates regression slope, significance, and explained deviance (R^2).

medRxiv preprint doi: https://doi.org/10.64898/2026.10.16.26365711; this version posted October 21, 2026. The copyright holder for this preprint (which was not certified by peer review) is the author/funder, who has granted medRxiv a license to display the preprint in perpetuity.
It is made available under a CC-BY 4.0 International license .

Supplementary Tables

Table S1. Characteristics of cells by quartile of cumulative releases per cell.

	**Quartile 1 (1262-6854 *w*Mel/cell)** N = 1,661	**Quartile 2 (6855-10428 *w*Mel/cell)** N = 1,661	**Quartile 3 (10429-14740 *w*Mel/cell)** N = 1,682	**Quartile 4 (14741-70074 *w*Mel/cell)** N = 1,637
**Intervention**		**Median** (IQR)		
*w*Mel introgression (%)	**31** (16, 50)	**27** (15, 44)	**24** (14, 42)	**21** (12, 32)
Trap count	**6.1** (3.6, 9.1)	**5.5** (3.2, 9.3)	**5.2** (3.2, 9.7)	**6.2** (3.7, 9.6)
**Socioeconomic**				
Inhabitants (0.01 km^2)	**85** (38, 136)	**119** (71, 205)	**110** (65, 171)	**101** (60, 137)
Income (R$/m)	**1,229** (829, 2,364)	**1,117** (768, 1,655)	**1,141** (770, 1,655)	**1,533** (1,205, 1,782)
**Environmental**				
Impervious surface (%)	**0.59** (0.35, 0.79)	**0.74** (0.57, 0.85)	**0.76** (0.62, 0.86)	**0.80** (0.70, 0.87)
Green space (%)	**0.018** (0.003, 0.056)	**0.005** (0.000, 0.021)	**0.003** (0.000, 0.014)	**0.002** (0.000, 0.008)
Elevation (m)	**13** (8, 30)	**16** (9, 37)	**12** (8, 24)	**14** (9, 21)

Table S2. Characteristics of cells by quartile of cumulative releases per inhabitant.

	**Quartile 1 (0-21 *w*Mel per inhabitant)** N = 1,898	**Quartile 2 (22-79 *w*Mel per inhabitant)** N = 1,898	**Quartile 3 (80-171 *w*Mel per inhabitant)** N = 1,897	**Quartile 4 (172-5714 *w*Mel per inhabitant)** N = 1,898
**Intervention**		**Median** (IQR)		
*w*Mel introgression (%)	**34** (12, 52)	**22** (14, 38)	**26** (16, 42)	**24** (13, 40)
Trap count	**5.4** (3.1, 8.4)	**5.4** (3.2, 8.6)	**5.8** (3.4, 9.5)	**5.7** (3.5, 10.3)
**Socioeconomic**				
Inhabitants (0.01 km^2)	**69** (17, 163)	**160** (115, 236)	**105** (80, 131)	**41** (20, 72)
Income (R$/m)	**1,297** (770, 2,631)	**1,021** (741, 1,555)	**1,492** (1,096, 1,928)	**1,385** (1,031, 1,754)
**Environmental**				
Impervious surface (%)	**0.55** (0.18, 0.78)	**0.75** (0.60, 0.85)	**0.74** (0.61, 0.83)	**0.76** (0.54, 0.86)
Green space (%)	**0.02** (0.00, 0.08)	**0.00** (0.00, 0.02)	**0.00** (0.00, 0.02)	**0.00** (0.00, 0.02)
Elevation (m)	**14** (8, 32)	**22** (9, 43)	**16** (10, 25)	**11** (8, 17)

medRxiv preprint doi: https://doi.org/10.64898/2026.10.16.26365711; this version posted October 21, 2026. The copyright holder for this preprint (which was not certified by peer review) is the author/funder, who has granted medRxiv a license to display the preprint in perpetuity.
It is made available under a CC-BY 4.0 International license .

Table S3. Characterization of GBMT clusters with covariates summarized by different radii around traps.

Characteristic	**Cluster 1** N = 203	**Cluster 2** N = 322	**Cluster 3** N = 275	**Cluster 4** N = 155	**p-value** *(Spearman-rank)*
	**Median** (IQR)				
**50 m radius**					
**Intervention**					
*w*Mel released (x1000)	**19** (4, 40)	**28** (10, 53)	**42** (23, 70)	**32** (14, 65)	<0.001
**Socioeconomic**					
Inhabitants (50m r)	**70** (5, 113)	**91** (40, 125)	**102** (57, 139)	**101** (54, 148)	<0.001
Income (R$/m)	**1,197** (826, 1,727)	**938** (659, 1,399)	**846** (671, 1,044)	**813** (593, 1,046)	<0.001
**Environmental**					
Impervious surface (%)	**61** (46, 74)	**74** (60, 84)	**81** (70, 87)	**82** (70, 89)	<0.001
Green space (%)	**1.40** (0.30, 3.81)	**0.40** (0.00, 1.62)	**0.12** (0.00, 0.71)	**0.13** (0.00, 0.74)	<0.001
Elevation (m)	**12** (7, 24)	**12** (8, 22)	**14** (10, 24)	**12** (9, 23)	0.018
**200 m radius**					
**Intervention**					
*w*Mel released (x1000)	**334** (133, 540)	**298** (190, 668)	**644** (387, 884)	**506** (314, 797)	<0.001
**Socioeconomic**					
Inhabitants (200m r)	**1,222** (127, 1,703)	**1,561** (793, 2,073)	**1,760** (1,214, 2,286)	**1,934** (1,171, 2,646)	<0.001
Income (R$/m)	**1,095** (801, 1,685)	**949** (677, 1,308)	**844** (645, 1,046)	**761** (564, 1,008)	<0.001
**Environmental**					
Impervious surface (%)	**57** (41, 67)	**70** (58, 81)	**78** (71, 84)	**79** (69, 85)	<0.001
Green space (%)	**2.25** (1.11, 4.90)	**1.14** (0.46, 2.46)	**0.46** (0.20, 1.14)	**0.47** (0.17, 1.48)	<0.001
Elevation (m)	**14** (8, 24)	**13** (9, 23)	**15** (10, 24)	**14** (9, 24)	0.07
**300 m radius**					
**Intervention**					
*w*Mel released (x1000)	**703** (255, 1,115)	**880** (373, 1,452)	**1,367** (817, 1,971)	**1,121** (665, 1,769)	<0.001
**Socioeconomic**					
Inhabitants (300m r)	**2,772** (387, 3,804)	**3,452** (2,206, 4,657)	**4,064** (2,994, 5,379)	**4,547** (2,991, 5,980)	<0.001
Income (R$/m)	**1,094** (760, 1,637)	**910** (652, 1,277)	**835** (655, 1,039)	**754** (553, 997)	<0.001
**Environmental**					
Impervious surface (%)	**55** (38, 66)	**69** (58, 78)	**77** (70, 82)	**77** (69, 82)	<0.001
Green space (%)	**2.27** (1.24, 4.85)	**1.41** (0.61, 2.61)	**0.58** (0.29, 1.28)	**0.57** (0.30, 1.52)	<0.001
Elevation (m)	**16** (8, 25)	**13** (9, 24)	**16** (10, 25)	**14** (9, 25)	0.116
**400 m radius**					
**Intervention**					
*w*Mel released (x1000)	**1,073** (420, 1,957)	**1,575** (638, 2,449)	**2,298** (1,406, 3,443)	**2,068** (1,247, 2,979)	<0.001
**Socioeconomic**					
Inhabitants (400m r)	**4,765** (1,044, 6,774)	**6,358** (4,084, 8,191)	**7,356** (5,749, 9,456)	**8,221** (5,841, 10,862)	<0.001
Income (R$/m)	**1,081** (771, 1,566)	**912** (664, 1,236)	**823** (642, 1,051)	**753** (560, 989)	<0.001
**Environmental**					
Impervious surface (%)	**55** (36, 65)	**68** (57, 77)	**76** (69, 80)	**77** (68, 81)	<0.001

medRxiv preprint doi: https://doi.org/10.64898/2026.10.16.26365711; this version posted October 21, 2026. The copyright holder for this preprint (which was not certified by peer review) is the author/funder, who has granted medRxiv a license to display the preprint in perpetuity.
It is made available under a CC-BY 4.0 International license .

Green space (%)	**2.25** (1.45, 4.84)	**1.58** (0.73, 2.64)	**0.70** (0.40, 1.39)	**0.77** (0.36, 1.58)	<0.001
Elevation (m)	**17** (8, 25)	**14** (9, 24)	**16** (10, 25)	**15** (10, 26)	0.121

Table S4. Variables tested as covariates in spatiotemporal models.

Type of Variable	Variable
**Intervention**	*w*Mel released (yes/no)
	*w*Mel released (log(1+x))
**Entomologic**	*w*Mel introgression (%)
	Ae. aegypti trap count (mosquitoes)
**Meteorologic**	Minimum weekly temperature (°C)
	Maximum weekly temperature (°C)
	Mean weekly temperature (°C)
	Mean daily precipitation (cm)
**Socioeconomic**	Mean income for persons >10 year of age (R$ per month)
	Mean head of household income (R$ per month)
	Proportion of persons earning minimum wage
	Proportion of persons earning half minimum wage
	Proportion of households with electricity
	Proportion of persons >5 years of age that are literate
	Proportion of households with waste management access
	Proportion of households with piped water access
	Population density (per 100m radius)

medRxiv preprint doi: https://doi.org/10.64898/2026.10.16.26365711; this version posted October 21, 2026. The copyright holder for this preprint (which was not certified by peer review) is the author/funder, who has granted medRxiv a license to display the preprint in perpetuity.
It is made available under a CC-BY 4.0 International license .

**Environmental**	Elevation (m)
	Impervious surface (%)
	Urban green space (%)
